# Enrichment and characterization of a haloalkaline-tolerant anammox bacterium

**DOI:** 10.1093/ismejo/wrag221

**Published:** 2026-08-27

**Authors:** Han-Yin Chuang, Jer-Horng Wu, Shan-Hua Chen, Wei-Yu Chen, Li-Jin Fong, Yi-Ting Lin

**Affiliations:** Department of Environmental Engineering, National Cheng Kung University, No.1, University Road, East District, Tainan City 70101, Taiwan; Department of Environmental Engineering, National Cheng Kung University, No.1, University Road, East District, Tainan City 70101, Taiwan; Department of Environmental Engineering, National Cheng Kung University, No.1, University Road, East District, Tainan City 70101, Taiwan; Department of Environmental Engineering, National Ilan University, No.1, Sec. 1, Shennong Rd., Yilan County, Yilan City 26047, Taiwan; Department of Environmental Engineering, National Cheng Kung University, No.1, University Road, East District, Tainan City 70101, Taiwan; Department of Environmental Engineering, National Cheng Kung University, No.1, University Road, East District, Tainan City 70101, Taiwan

**Keywords:** *Candidatus* Loosdrechtia, anammox taxonomy, saline-alkaline stress, pH homeostasis, Mrp cation/proton antiporter

## Abstract

Metagenomic surveys have substantially expanded the known diversity of anaerobic ammonium-oxidizing (anammox) bacteria. However, the physiological traits of newly proposed anammox genera remain largely hypothetical due to the lack of cultured representatives. Although niche differentiation driven by salinity, organic substrates, and oxygen is well documented in anammox bacteria, adaptations to alkaline environments remain uncharacterized. Moreover, no anammox lineage has been identified as an alkaline specialist. Herein, we report the enrichment (>80% relative abundance) of an anammox bacterium, provisionally named *Candidatus* Loosdrechtia alkalitolerans, which represents the cultured member of the genus *Ca*. Loosdrechtia. *Ca*. L. alkalitolerans exhibits marked haloalkaline tolerance, outcompeting other freshwater anammox genera under long-term saline-alkaline stress conditions (0.5% NaCl; pH ~ 9.13). Comparative genomics revealed that among freshwater anammox lineages, only *Ca.* L. alkalitolerans encodes a complete multiple resistance and pH adaptation (Mrp) cation/proton antiporter operon. Batch assays with transcriptional profiling demonstrated that sodium addition upregulated *mrp* expression and recovered anammox activity under alkaline stress, suggesting that Mrp-mediated cation/proton exchange may contribute to pH homeostasis in *Ca*. L. alkalitolerans. Furthermore, database mining revealed that the habitat of *Ca.* L. alkalitolerans is not restricted to haloalkaline environments, but extends to diverse freshwater and wastewater ecosystems. These findings provide mechanistic insight into saline-alkaline tolerance in anammox bacteria, expanding understanding of their physiological plasticity and ecological roles.

## Graphical Abstract

**Figure:**
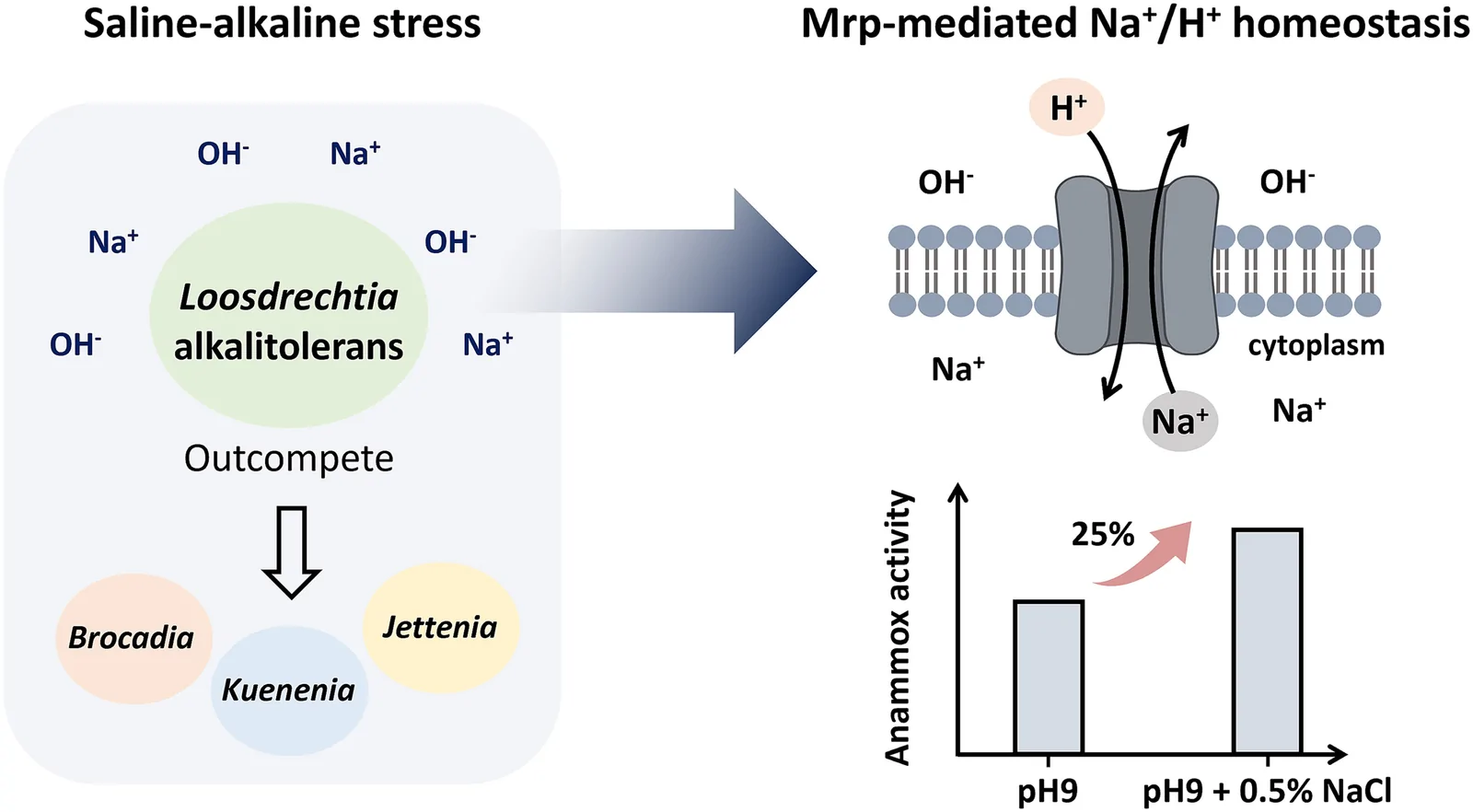

## Introduction

Anaerobic ammonium oxidation (anammox) is a key component of the global nitrogen cycle and a vital pathway for sustainable wastewater treatment [1, 2]. This process is mediated by a monophyletic group of as-yet-isolated bacteria within the phylum *Planctomycetes*. Recent metagenomic surveys have revealed extensive diversity within this guild, expanding the clade to > 12 proposed genera [3]. However, physiological and ecological understanding in this field lags behind genomic discovery. To date, cultivation success has been limited to species within *Candidatus* Kuenenia, *Candidatus* Brocadia, *Candidatus* Jettenia, *Candidatus* Anammoxoglobus, and *Candidatus* Scalindua [4, 5]. The metabolic versatility and stress adaptation mechanisms of most anammox lineages, particularly those known only from environmental sequences [6–10], remain hypothetical.

Ecophysiological characterization of cultivated anammox species has revealed distinct niche differentiation driven by substrate kinetics and environmental stressors [5, 11]. Marine *Ca*. Scalindua sp. exhibits high salinity and oxygen tolerance [12, 13], but shows a lower growth rate and biomass yield than freshwater anammox lineages [14]. *Ca*. Brocadia can rapidly proliferate in substrate-rich environments [15], but its relatively high maintenance energy requirement contributes to lower resilience to environmental stressors [12, 13, 16]. In contrast, *Ca*. Kuenenia stuttgartiensis shows higher affinities for NH_4_^+^ and NO_2_^−^ than *Ca*. Brocadia sinica and *Ca*. Jettenia caeni and can grow across a broader salinity range [17]. However, intrinsic adaptation to alkaline environments has not been demonstrated in anammox bacteria. Although anammox granular sludge can withstand pH shocks (pH 9–10.5) [18–20], this resilience has been attributed to mass-transfer limitations within the granule matrix that buffer the local microenvironment rather than to cellular adaptation. Conversely, planktonic enrichment cultures are generally neutrophilic (6.8–8.5) [5] and typically lose activity above pH 9 in batch assays [14, 21–23], reflecting the difficulty of maintaining cytoplasmic pH homeostasis under alkaline conditions. This challenge is exacerbated in ecosystems characterized by simultaneous saline and alkaline stress, such as industrial wastewater [24–26], in which ion imbalances impose an additional maintenance energy burden. Whether specific anammox lineages have evolved in response to these stress conditions remains unclear.

Herein, we report the enrichment of an anammox bacterium, provisionally named *Candidatus* Loosdrechtia alkalitolerans, obtained through long-term reactor operation. Ecological succession among anammox bacteria in the reactor delineated the realized niche of *Ca.* L. alkalitolerans under saline-alkaline stress conditions. Physiological assays, comparative genomics, and metatranscriptomic profiling further suggested cation-coupled pH homeostasis mediated by a multiple resistance and pH adaptation (Mrp) cation/proton antiporter complex in *Ca.* L. alkalitolerans. These findings provide mechanistic evidence of saline-alkaline tolerance in anammox bacteria and expand our understanding of their ecological versatility.

## Materials and methods

### Reactor operation

Activated sludge collected from a full-scale petrochemical wastewater treatment plant in Tainan City, Taiwan, was used as the inoculum. Amplicon sequencing revealed the presence of *Ca.* L. alkalitolerans in this sludge (see Results section). Three 3-L sequencing batch reactors were sequentially established to investigate how combined environmental stressors affect anammox consortia.

Glutamate has been reported as a key compatible solute in anammox bacteria, and the addition of exogenous glutamate has been shown to improve reactor performance [13, 27]. We therefore examined whether glutamate supplementation differentially affects anammox consortia under saline and freshwater conditions. The freshwater reactor (FGA) was operated without sodium chloride (NaCl) supplementation, whereas the saline reactor (SGA) was operated with NaCl gradually increased from 0.5% to 3.5% (w/v) and subsequently decreased to 0.5%. Both reactors were continuously fed with monosodium L-glutamate. An initial concentration of 0.2 mM was applied to prevent excessive growth of heterotrophs, which was later increased to 1 mM in the SGA reactor to evaluate its potential to alleviate severe osmotic stress [13, 27]. Both reactors were maintained at room temperature in the dark with magnetic stirring at 180 rpm. After 310 days of operation, sludge from the FGA, SGA, and other anammox reactors in our laboratory [27, 28] was combined to inoculate a new reactor, called KJ. This reactor was fed with influent containing 0.5% (w/v) NaCl without glutamate. The operational parameters of the three reactors at different stages and the composition of the synthetic medium are provided in Tables S1 and S2. Unless otherwise specified, salinity (%) refers to the additional NaCl incorporated into the medium. Nitrogen species were quantified using colorimetric methods and ion chromatography, and pH was measured using a benchtop pH meter. Details regarding these analyses and the reactor performance evaluation are provided in Text S1.

### Short-term batch assays

Before the experiments, the KJ reactor was operated without additional NaCl, and an effluent pH of 8.52 ± 0.14 was maintained for 4 months (Table S1 and Fig. S1). At the end of this period (Day 737), 16S rRNA gene amplicon sequencing was performed. The results indicated that *Ca.* L. alkalitolerans constituted 66% of the total bacterial community (Fig. S2). For all batch assays, a 30-ml aliquot of biomass (1.22–1.39 g-volatile suspended solids [VSS]/L) was transferred directly from the KJ reactor into 125 ml serum bottles by using a peristaltic pump under continuous N_2_ purging and then sealed with butyl rubber stoppers and aluminium caps. For each batch assay, all treatments were prepared in triplicate on the same day and incubated at 35°C and 150 rpm in the dark for 8–12 h. Specific anammox activity (SAA) was calculated from the linear regression of the total ammonium nitrogen (NH_3_ + NH_4_^+^) depletion profile (*R*^2^ > 0.84 for all assays) and normalized to the VSS concentration in each bottle. VSS was determined following American Public Health Association (APHA) Standard Method 2540E. Differences in SAA and gene expression were assessed using Student’s *t* test in Excel. A *P* value of <0.05 indicated significance.

#### pH-dependent SAA

The medium contained 36.4 ± 5.0 mg-N/L NH_3_ + NH_4_^+^ and 56.5 ± 2.2 mg-N/L nitrite (NO_2_^−^) as anammox substrates. Initial pH values were adjusted to 7.0, 8.0, 8.5, 9.0, 9.5, and 10.0 with 0.05 N hydrogen chloride or 0.05 N sodium hydroxide and measured using pH test strips (Rapid Tests pH-Fix 4.5–10.0; Macherey-Nagel, Germany). pH was not further adjusted during incubation.

#### Salinity-dependent SAA

The medium contained 71.4 ± 3.4 mg-N/L NH_3_ + NH_4_^+^ and 84.0 ± 4.0 mg-N/L NO_2_^−^ as anammox substrates. The initial pH was set to 8.12 for all bottles, and salinity was adjusted to 0%, 0.5%, 1%, 2%, 3%, and 4% (w/v) with NaCl.

#### Effects of sodium on SAA and Mrp expression under alkaline conditions

To evaluate the combined effect of saline-alkaline stress on *Ca.* L. alkalitolerans, batch assays were conducted under three conditions: baseline, pH 8 (optimal condition); alkaline control, pH 9 (half-maximal inhibitory condition); and Na^+^-supplemented alkaline treatment, pH 9 with 0.5% (w/v) NaCl. The medium contained 46.1 ± 2.3 mg-N/L NH_3_ + NH_4_^+^ and 54.1 ± 1.6 mg-N/L NO_2_^−^ as anammox substrates. pH was not further adjusted during incubation. At the end of incubation, triplicate biomass samples were harvested for RNA extraction and subsequent metatranscriptomic analysis. The relative transcript levels of *mrp* genes specific to *Ca.* L. alkalitolerans were further confirmed by reverse transcription quantitative polymerase chain reaction (RT-qPCR) using the 2^−∆∆Ct^ method, with the 16S rRNA gene of *Ca.* L. alkalitolerans as the internal control (Text S2 and Table S3).

### Amplicon sequencing and analysis

The V3–V4 hypervariable region of the 16S rRNA gene was amplified using the universal bacterial primer pair 341F/805R and sequenced on an MiSeq platform (2 × 300 bp; Illumina, USA). Amplicon sequence variants (ASVs) were generated using Qiime2 v2024.10 [29], and taxonomic assignment was performed against the SILVA 138.2 SSURef_NR99 database (Text S3).

### Metagenomic and metatranscriptomic sequencing and analysis

A genomic DNA sample collected from the KJ reactor on Day 339 and RNA samples collected in triplicate at the end of the batch assays under pH 8, pH 9, and pH 9 with 0.5% NaCl conditions were sequenced on the NovaSeq platform (2 × 150 bp; Illumina) by BIOTOOLS (Taiwan). Metagenome-assembled genomes (MAGs) were generated following a previously described workflow and parameters [30]. Briefly, raw reads were trimmed with Trimmomatic v0.39 [31] and *de novo* assembled using Megahit v1.2.9 [32]. Binning was performed using CONCOCT v0.4.0 [33], MaxBin2 v2 [34], and MetaBAT2 v2.12.1 [35], followed by refinement with MetaWRAP v1.3.2 [36]. For metatranscriptomic analysis, raw RNA reads were filtered using Trimmomatic v0.39, and rRNA reads were removed using SortMeRNA v4.3.6 against the smr_v4.3_default database [37]. The remaining non-rRNA reads were used for subsequent gene expression analysis.

### Genome annotation

Two putative anammox MAGs (JSbin17 and JSbin29) were identified using GTDB-Tk v2.4.0 against the GTDB R220 release [38]. The MAG JSbin17, whose completeness and contamination were estimated at 98.9% and 1.65%, respectively, by CheckM v1.2.4 [39], represents the anammox bacterium *Ca.* L. alkalitolerans proposed in this study. Open reading frames (ORFs) were predicted using Prokka v1.14.6 [40] and annotated through searches against the Kyoto Encyclopedia of Genes and Genomes (KEGG) GENES and eggNOG databases using GhostKOALA [41] and eggNOG-mapper v2.1.12 [42], respectively. COG functional categories were assigned based on eggNOG-derived COG IDs using the NCBI COG2024 database. In addition, the ORFs were searched against the NCBI non-redundant database, Transporter Classification Database (TCDB) [43], and a custom database comprising anammox-associated proteins and Mrp antiporter subunits (Supplementary Data Sheet 1) using DIAMOND blastp v2.1.10 [44] with an e-value cutoff of <10^−10^. To distinguish Mrp antiporter subunits from homologous respiratory chain complex I subunits [45], putative hits were further examined for their co-localization within predicted operons using Operon-mapper [46], and visualized using clinker and clustermap.js v0.0.32 [47].

### Taxonomic, phylogenetic, and environmental distribution analyses

The *Ca.* L. alkalitolerans MAG was analysed together with 36 reference anammox MAGs retrieved from the NCBI Genome database (accessed August 2025; Table S4). To assess taxonomic relatedness, full-length 16S rRNA gene sequences were aligned and used to generate an identity matrix by Clustal Omega v1.2.4 [48]. Digital DNA–DNA hybridization (dDDH), orthologous average nucleotide identity (OrthoANI), average amino acid identity (AAI), and percentages of conserved proteins (POCP values) were calculated using Genome-to-Genome Distance Calculator 3.0 [49], PyOrthoANI v0.7.0 [50], EzAAI v1.2.4 [51], and POCP-nf pipeline v2.3.6 [52], respectively. For phylogenetic analyses, anammox-affiliated ASVs from amplicon sequencing and full-length 16S rRNA genes extracted from the anammox MAGs were aligned with their respective reference sequences using Muscle5 v5.3 [53] with default settings. A total of 74 bacterial single-copy genes were identified and aligned using the built-in *Bacteria* single-copy gene HMM set in GToTree v1.6.31 workflow and its dependency programs [54]. Hydrazine dehydrogenase (HDH), MrpA, and MrpD amino acid sequences were extracted from the anammox MAGs and aligned with reference sequences using MAFFT v7.525 with the L-INS-i option [55]. All above alignments were trimmed using trimAl v1.5.0 with the -automated1 option [56]. Maximum-likelihood trees were then constructed using IQ-TREE3 v3.0.1 [57] under the best-fit models selected by ModelFinder, with 1000 ultrafast bootstrap replicates, and visualized in iTOL v7 [58]. The global occurrence of *Ca.* L. alkalitolerans was investigated using IMNGS [59] with a 95% similarity threshold and a minimum sequence length of 200 bp (Text S4).

### Gene expression profiling

To compare the gene expression profile of *Ca.* L. alkalitolerans under pH 8, pH 9, and pH 9 with NaCl, the non-rRNA reads were pseudoaligned against the predicted ORFs of *Ca.* L. alkalitolerans MAG, and transcript abundances were estimated as transcripts per million (TPM) using kallisto v0.52.0 [60] with 100 bootstrap replicates. DESeq2 v1.46.0 [61] was used to estimate log_2_ fold changes among conditions, which were subsequently used for pre-ranked gene set enrichment analysis (GSEA) with clusterProfiler v4.14.6 [62] in R v4.4.1. COG functional categories represented by more than 5 ORFs were used as gene sets, and functional enrichment was evaluated based on normalized enrichment scores (NESs). Adjusted *P* values were calculated using the Benjamini–Hochberg method, with values <0.05 considered significant.

## Results

### Temporal dynamics of anammox bacteria in response to salinity, organic substrates, and alkalinity

To decouple the effects of organic substrates and salinity on anammox communities, two sequencing batch reactors were operated with continuous glutamate feeding under freshwater (FGA) and varying salinity (SGA) conditions. The FGA reactor maintained stable performance throughout the 310-day operation (Figs. S3A and S3B). Amplicon sequencing of the inoculum revealed a community dominated by AMX_ASV2 (5.1%), classified as *Ca.* K. stuttgartiensis. A second lineage, AMX_ASV1 (1.1%), was assigned to an unclassified member of *Ca*. Brocadiaceae and is hereafter designated as *Brocadiaceae* ASV1. Under freshwater conditions with continuous glutamate feeding, the diversity of anammox bacteria increased during succession. *Ca.* K. stuttgartiensis and Three *Ca*. Brocadia-affiliated ASVs (AMX_ASV3-AMX_ASV5) increased in abundance, and *Ca*. Jettenia caeni emerged (AMX_ASV6; 0.7%). By contrast, *Brocadiaceae* ASV1 declined to 0.9%. These results suggest that glutamate supplementation favours *Ca*. Brocadia and *Ca*. Kuenenia over *Brocadiaceae* ASV1 in freshwater environments.

**Figure 1:**
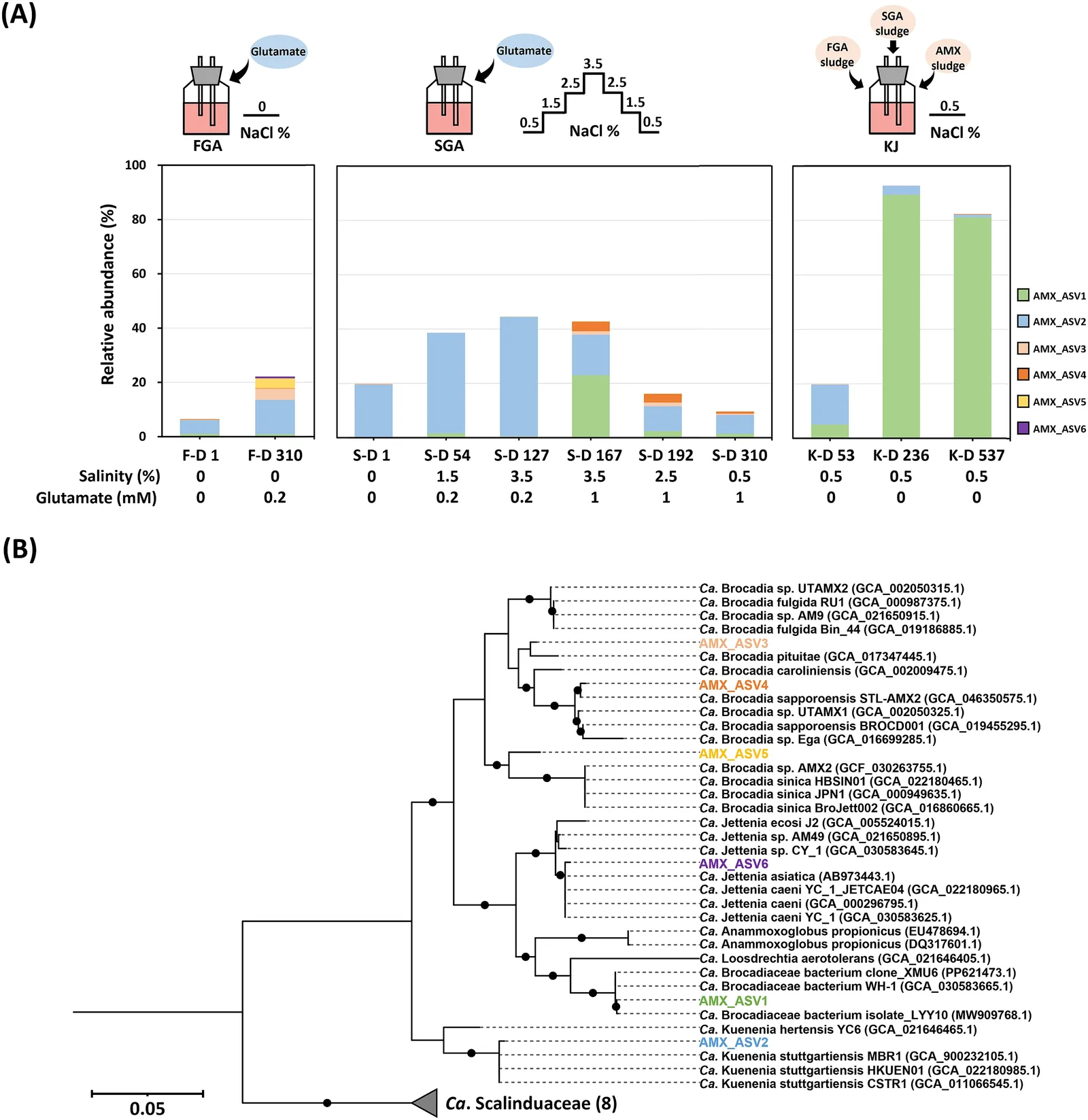
Temporal succession and phylogenetic placement of anammox bacteria. (A) Relative abundances of anammox-affiliated amplicon sequence variants (ASVs) during the operation of the FGA, SGA, and KJ reactors. The sampling day, salinity (% NaCl, w/v), and glutamate concentration (mM) for each sample are detailed below the bars. (B) Maximum-likelihood phylogenetic tree of the anammox-affiliated ASVs based on the V3–V4 region of the 16S rRNA gene (ca. 403 bp). The tree was inferred using IQ-TREE3 under the best-fit model TPM3u + I + G4, with 1000 ultrafast bootstrap replicates; bootstrap values >90 are shown in solid circles. The scale bar shows the estimated number of substitutions per site. The *Ca*. Scalinduaceae clade (n = 8) was collapsed and used as the outgroup. ASVs detected in this study are highlighted using the same colors as in panel (A).

In the SGA reactor, nitrogen removal remained robust at low salinity (0.5%–1.5% NaCl), with *Ca.* K. stuttgartiensis (19.5%–37.0%) dominating the community (Fig. 1A). Performance declined at 2.5% salinity and collapsed at 3.5%, despite *Ca.* K. stuttgartiensis reaching a relative abundance of 44.0% (Figs. S3C and S3D). Increasing glutamate to 1 mM (Day 127), a concentration previously shown to enhance reactor performance under salinity inhibition [27], did not restore activity under severe osmotic stress. Although the nitrogen removal efficiency recovered to 74.7% ± 12.1% after salinity was gradually reduced to 0.5%, it remained significantly lower than the pre-inhibition values (*P* < 0.01), indicating a persistent legacy effect of osmotic shock. As salinity declined, *Ca*. Brocadia (AMX_ASV3 and AMX_ASV4) re-emerged; however, *Ca.* K. stuttgartiensis generally maintained dominance over *Brocadiaceae* ASV1, which confirmed its competitive advantage under saline conditions.

To reduce the labor required to maintain multiple anammox systems, sludge from the FGA, SGA, and *Ca*. Kuenenia-dominant reactors [27, 28] was combined to inoculate the KJ reactor. This reactor was operated at 0.5% NaCl without glutamate (Table S1). Operation remained stable as the total nitrogen loading rate increased (Fig. S3E and S3F). The influent pH was set at 7.37 ± 0.17, whereas the reactor pH was not externally controlled and increased to 9.13 ± 0.25 by the end of each cycle (Fig. S4B) due to alkalinity generated by the anammox reaction. This pH value exceeded the optimal growth range of most cultivated anammox bacteria [4, 5]. Although the effluent pH approached the NH_3_/NH_4_^+^ p*K*a, free ammonia remained below 20 mg-N/L under the SBR operation mode (Fig. S4C), and the linear total ammonia nitrogen depletion profile within a single cycle indicated no apparent inhibition (Fig. S4D). Under these saline-alkaline conditions, the community exhibited a marked shift. By Day 236, *Brocadiaceae* ASV1 had outcompeted *Ca.* K. stuttgartiensis and all other anammox lineages, reaching a relative abundance of >80% (Fig. 1A). This dominance persisted through Day 537. By contrast, the relative abundance of *Ca.* K. stuttgartiensis declined to <1.0%. These results suggest that although *Ca*. Kuenenia prevails under salinity stress, saline-alkaline stress confers a selective advantage to *Brocadiaceae* ASV1.

### Salinity- and pH-dependent SAA of *Brocadiaceae* ASV1-dominant sludge

To delineate the short-term response of the *Brocadiaceae* ASV1-dominant enrichment, SAA was quantified across pH gradients (7–10) and salinity levels (0%–4% w/v NaCl) during 8–12 h incubations. The biomass used in both assays was collected from the KJ reactor, which exhibited stable performance over 4 months of operation without additional NaCl, with an effluent pH of 8.52 ± 0.14 (Table S1 and Fig. S1).

**Figure 2:**
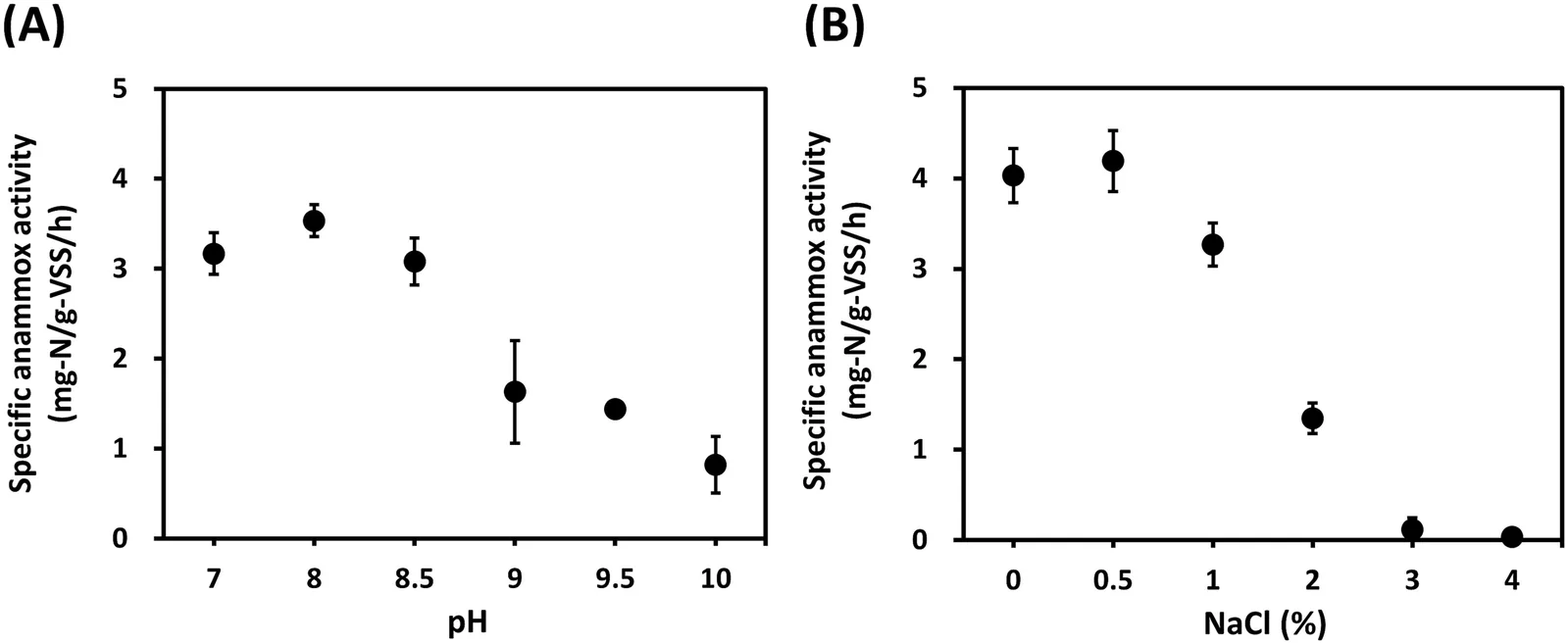
Haloalkaline tolerance of the *Ca*. Loosdrechtia alkalitolerans enrichment. Specific anammox activity (SAA) of the dominant sludge was evaluated under varying pH (A) and NaCl (B) conditions. Biomass (1.39–1.55 g-VSS/L) from the KJ reactor was used as the inoculum for both batch assays. SAA was calculated from the linear regression of the NH_3_ + NH_4_^+^ depletion profile (*R*^2^ > 0.84) and normalized to VSS. Error bars represent the standard deviation from three biological replicates.

The highest SAA was observed at pH 8 and retained >80% of the maximum activity between pH 7.0 and 8.5 (Fig. 2A). The enrichment exhibited pronounced alkaline tolerance, retaining ~50% of the maximum activity at pH 9.0–9.5 and 20% at pH 10. This tolerance contrasts with previously characterized planktonic anammox enrichments, which typically lose activity completely above pH 9.0 (Table S5). Regarding salinity, SAA was optimal at 0%–0.5% NaCl, decreased to 33% at 2% NaCl, and was virtually undetectable at >3% NaCl (Fig. 2B). These results indicate that *Brocadiaceae* ASV1 can adapt to alkaline environments with moderate salinity tolerance.

### Phylogenetic and taxonomic position of *Brocadiaceae* ASV1

**Figure 3:**
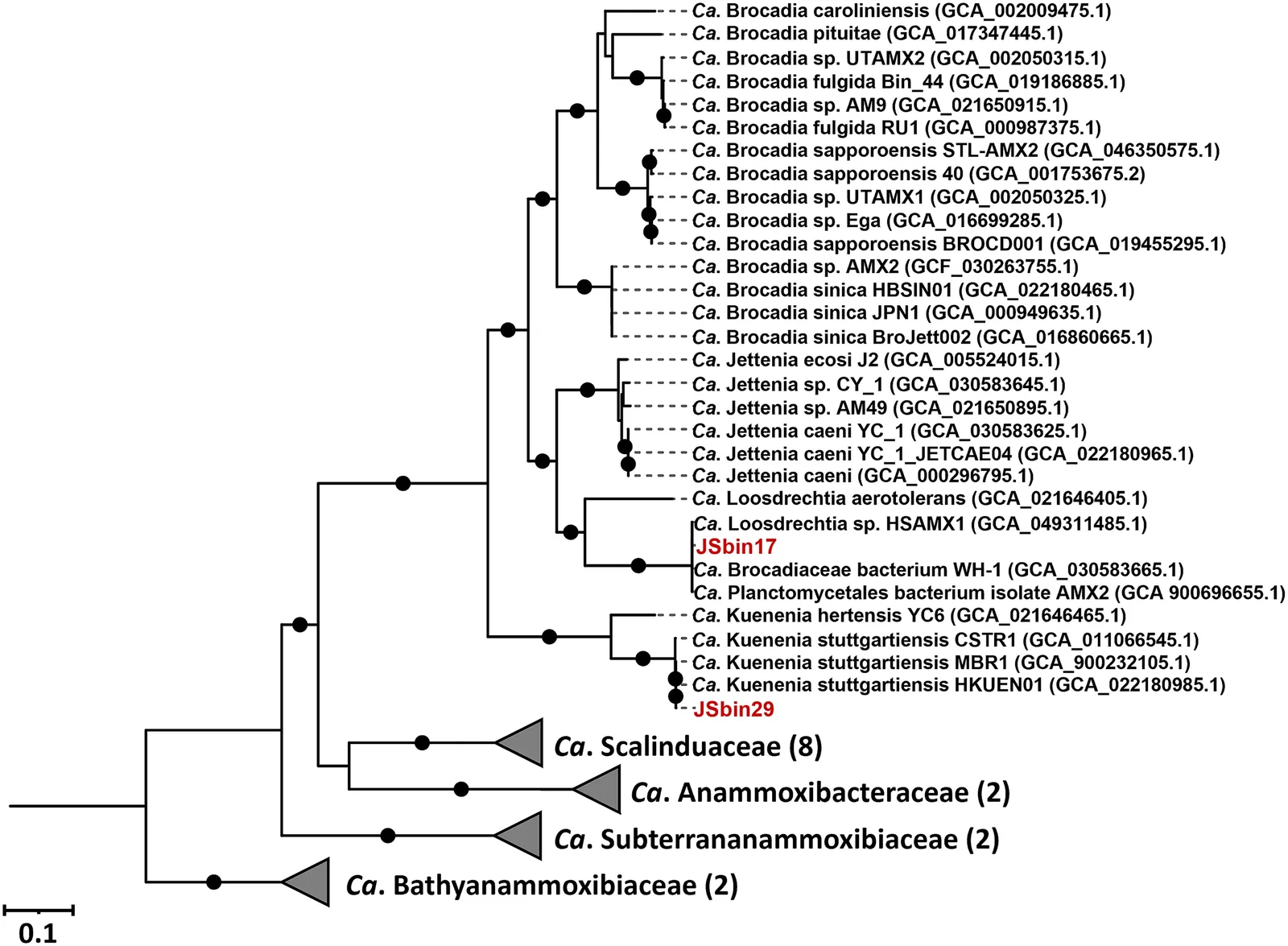
Phylogenomic placement of anammox bacteria based on concatenated single-copy marker genes. A maximum-likelihood tree was constructed from 74 bacterial single-copy genes. The tree was inferred using IQ-TREE3 under the best-fit model Q.PLANT+F + I + R4 selected by ModelFinder, with 1000 ultrafast bootstrap replicates; bootstrap values >90 are shown in solid circles. Deep-branching clades were collapsed, with the number of sequences in each clade indicated in parentheses. The *Ca*. Bathyanammoxibiaceae clade was used as the outgroup. The scale bar shows the estimated number of substitutions per site. The anammox genomes reconstructed in this study (JSbin17 and JSbin29) are highlighted in red.

To determine the taxonomic identity of *Brocadiaceae* ASV1, metagenomic analysis was performed on high-quality MAGs recovered from KJ reactor sludge (Day 339). Two *Brocadiaceae*-affiliated MAGs were identified (Table S6). The MAG JSbin29 was identified as *Ca.* K. stuttgartiensis (ANI: 99.6%; 16S rRNA gene identity to AMX_ASV2: 100%), whereas the MAG JSbin17 shared 100% 16S rRNA gene identity with the dominant *Brocadiaceae* ASV1. Phylogenomic reconstruction based on 74 bacterial single-copy marker genes (Fig. 3), full-length 16S rRNA genes (Fig. S5A), and amino acid sequences of HDH (Fig. S5B) consistently placed JSbin17 with several uncharacterized MAGs (WH-1, HSAMX1, and AMX2), forming a distinct clade sister to *Ca.* Loosdrechtia aerotolerans.

To compare JSbin17 with known anammox bacteria, full-length 16S rRNA gene identity and several overall genomic relatedness indices (OGRIs) were analyzed. JSbin17 shared higher full-length 16S rRNA gene identity with *Ca*. Jettenia spp. (91.9%–92.7%) than with *Ca*. L. aerotolerans (91.6%; Fig. S6). In contrast, OGRIs analyses identified *Ca*. L. aerotolerans as its closest described relative (Figs. S7 and S8). The AAI and POCP values between JSbin17 and *Ca*. L. aerotolerans (74.8% and 62.9%, respectively) fell within accepted genus-level ranges (AAI: 65%–95%; POCP: >50%) [63, 64], whereas the OrthoANI (75.0%) and dDDH (22.0%) values were well below the species delineation thresholds (95%–96% for OrthoANI and 70% for dDDH) [49, 65]. Despite discrepancies in 16S rRNA gene identity patterns, the genomic distinctiveness and consistent phylogenomic placement of JSbin17 support its assignment as a distinct species within the genus *Ca*. Loosdrechtia. Therefore, JSbin17 was provisionally named “*Candidatus* Loosdrechtia alkalitolerans.”

### Genomic potential of *Ca.* L. alkalitolerans for anammox metabolism and stress adaptation

**Figure 4:**
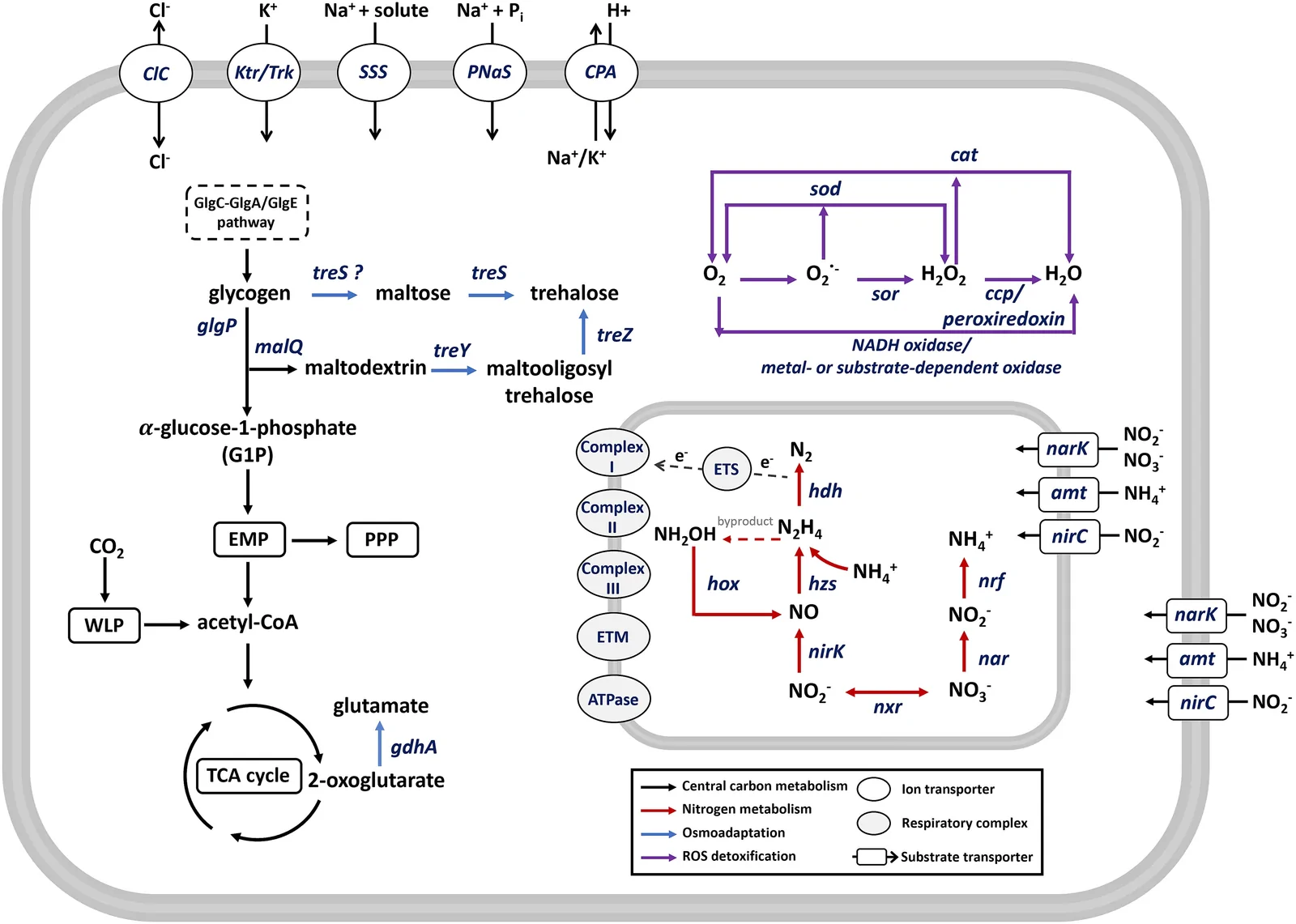
Overview of central anammox metabolism and stress-response pathways in *Ca.* Loosdrechtia alkalitolerans. Pathways for central carbon metabolism, nitrogen metabolism, osmoadaptation, and reactive oxygen species (ROS) detoxification are shown by black, red, blue, and purple arrows, respectively. Abbreviations: EMP, Embden-Meyerhof-Parnas pathway; PPP, pentose phosphate pathway; WLP, Wood-Ljungdahl pathway; TCA cycle, tricarboxylic acid cycle; ETS, electron transfer shuttle proteins; ETM, electron transfer module. Genes encoding the enzymes involved in each reaction are detailed in Supplementary Data Sheet 2.

The genome of *Ca.* L. alkalitolerans encodes the complete metabolic repertoire required for anammox reaction (Fig. 4 and Supplementary Data Sheet 2). Consistent with *Ca.* L. aerotolerans [6] and *Ca*. Jettenia spp. [66–68], *Ca.* L. alkalitolerans likely utilizes a NirK-type copper-containing nitrite reductase to reduce nitrite to nitric oxide, followed by hydrazine formation mediated by the hydrazine synthase (HZS). In addition, seven homologs of hydroxylamine oxidoreductase (HAO)-like proteins from *Ca.* K. stuttgartiensis were identified (amino acid sequence identity: 51.6%–88.5%), including hydrazine dehydrogenase (HDH), hydroxylamine oxidase (HOX), and reductive HAO, which has been proposed to reduce nitrite to nitric oxide [69, 70]. The genome of *Ca.* L. alkalitolerans also encodes a nearly complete electron transport chain and key electron transfer shuttle proteins (KsTH, Kustc0563, NaxL, NaxS, and NXRT), which may facilitate electron flow between soluble and membrane-bound complexes [71–74].

For saline-alkaline stress adaptation, *Ca.* L. alkalitolerans encodes diverse ion transport systems, including potassium uptake channels (Ktr and TrK families), chloride channels (ClC family), monovalent cation/proton antiporters (CPA family), and Na^+^-coupled symporters (SSS and PNaS families) for maintaining ion and pH homeostasis. In addition to ion transport, glutamate and trehalose, key osmolytes in anammox bacteria [13], can be synthesized from 2-oxoglutarate by glutamate dehydrogenase and from maltodextrin through the TreY/TreZ or TreS pathways, respectively. Given that saline-alkaline stress can induce oxidative stress [75–77], *Ca.* L. alkalitolerans encodes several antioxidant enzymes, including superoxide dismutase–catalase and superoxide reductase–cytochrome *c* peroxidase systems. A putative bilirubin oxidase capable of reducing O_2_ to H_2_O was also identified, a trait shared with *Ca.* L. aerotolerans [6]. Together, these genomic features suggest a specialized adaptation strategy that enables *Ca.* L. alkalitolerans to persist in saline-alkaline environments.

### Comparative genomic and phylogenetic analyses of cation/proton antiporters in anammox bacteria

**Figure 5:**
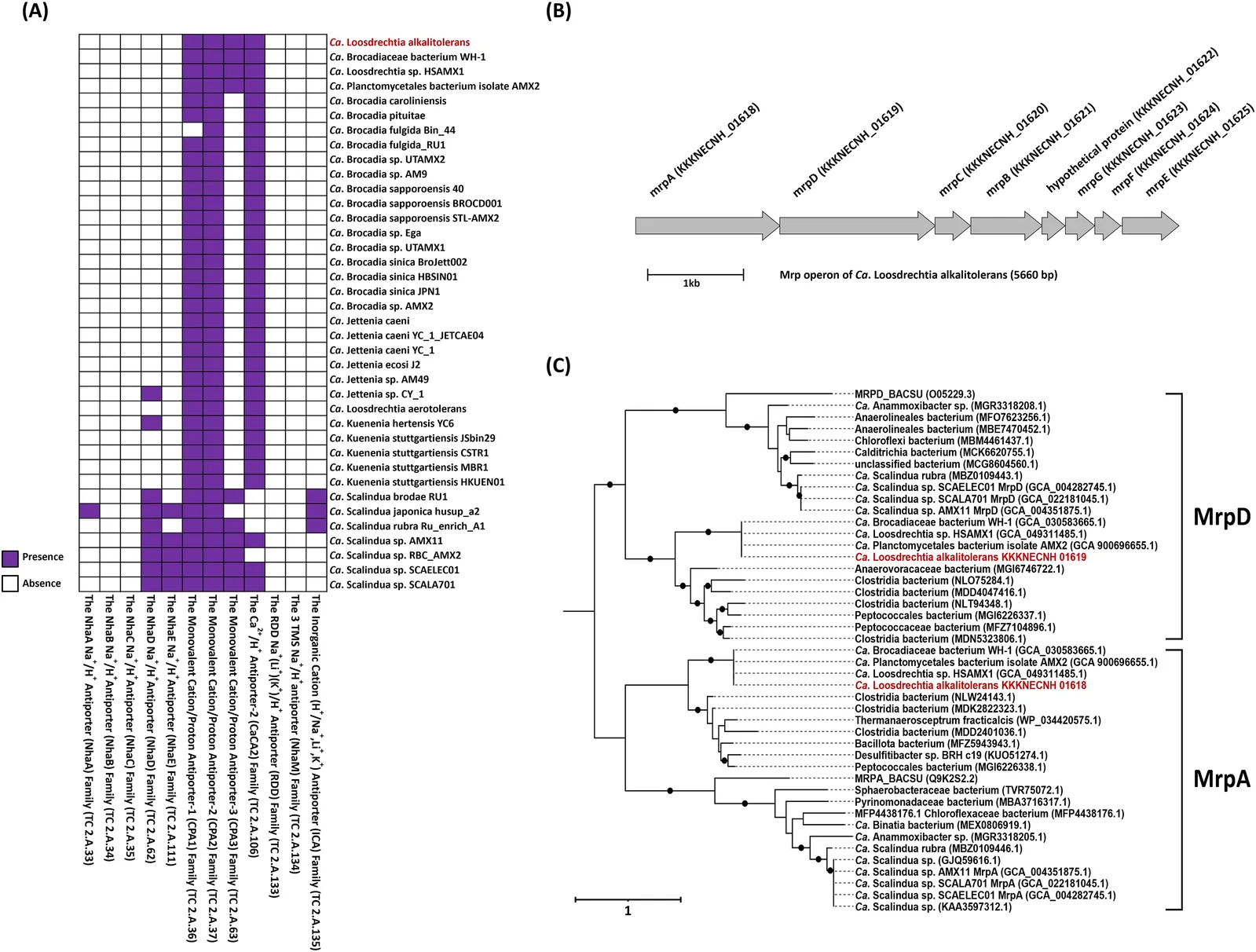
Comparative genomic and phylogenetic analyses of cation/proton antiporters in anammox bacteria. (A) Distribution of cation/proton antiporter families across *Ca.* Loosdrechtia alkalitolerans and 36 publicly available anammox metagenome-assembled genomes (MAGs) based on transporter classification database (TCDB) annotations. The heatmap indicates the presence and absence of TC families in purple and white, respectively. (B) Organization of the Mrp operon (5660 bp) in *Ca.* Loosdrechtia alkalitolerans. Numbers in parentheses denote the locus tags of the open reading frames. (C) Maximum-likelihood phylogenetic tree of MrpA and MrpD amino acid sequences from *Ca*. Loosdrechtia alkalitolerans, *Ca*. Scalindua spp., and representative reference bacteria. The tree was inferred using IQ-TREE3 under the best-fit model LG + F + G4 selected by ModelFinder, with 1000 ultrafast bootstrap replicates; bootstrap values >90 are shown in solid circles. The scale bar shows the estimated number of substitutions per site. The sequences of *Ca.* Loosdrechtia alkalitolerans are highlighted in red.

Cation/proton antiporters that mediate H^+^ influx in exchange for cations play an important role in ion and pH homeostasis. To compare this capacity among anammox genera, we profiled cation/proton antiporter families across the anammox bacteria by using TCDB annotations (Fig. 5A). The CPA1, CPA2, and CaCA2 families are conserved across all examined MAGs, suggesting a shared core toolkit for basal cation/proton balance. Beyond this conserved core, antiporter repertoires differed among anammox lineages. The marine genus *Ca*. Scalindua has more antiporters than does the primarily freshwater family *Ca*. Brocadiaceae, reflecting niche specialization where high salinity requires a broad range of ion exchange mechanisms. Variation was also observed within the freshwater family *Ca*. Brocadiaceae. *Ca*. Jettenia sp. CY_1 and *Ca*. Kuenenia hertensis YC6 encode the NhaD family. Although NhaD confers salt resistance, its role in pH homeostasis appears to be limited [78]. By contrast, *Ca.* L. alkalitolerans and its closely related MAGs (WH-1, HSAMX1, and AMX2) uniquely encode the CPA3 family (Mrp complex), a system central to concurrent salinity tolerance and alkaline pH homeostasis in diverse prokaryotes [78–81]. Because Mrp function is contingent upon the assembly of a multi-subunit complex [82–84], we verified the presence of a complete eight-gene operon in *Ca.* L. alkalitolerans (Fig. 5B). This complete system is absent in all other *Ca*. Brocadiaceae genomes analysed, which provides a genomic basis for the resilience and dominance of *Ca.* L. alkalitolerans in the saline-alkaline KJ reactor. Although complete Mrp operons were also identified in marine *Ca*. Scalindua spp. (AMX11, SCAELEC01, and SCALA701; recently proposed as the new genus *Ca*. Actiscalindua [10]) and *Ca*. Anammoxibacter, phylogenetic analysis revealed a distinct evolutionary origin. The Mrp subunits of *Ca.* L. alkalitolerans shared low amino acid sequence identity (22.0%–33.1%) with marine homologs and formed a separate cluster in the phylogenetic tree (Figs. 5C and S9). The clustering with *Clostridia* sequences suggests that *Ca.* L. alkalitolerans likely acquired the Mrp complex through horizontal gene transfer from *Firmicutes* rather than inheriting it vertically from the ancestral marine anammox lineage [85].

### Effects of sodium on anammox activity under alkaline conditions

**Figure 6:**
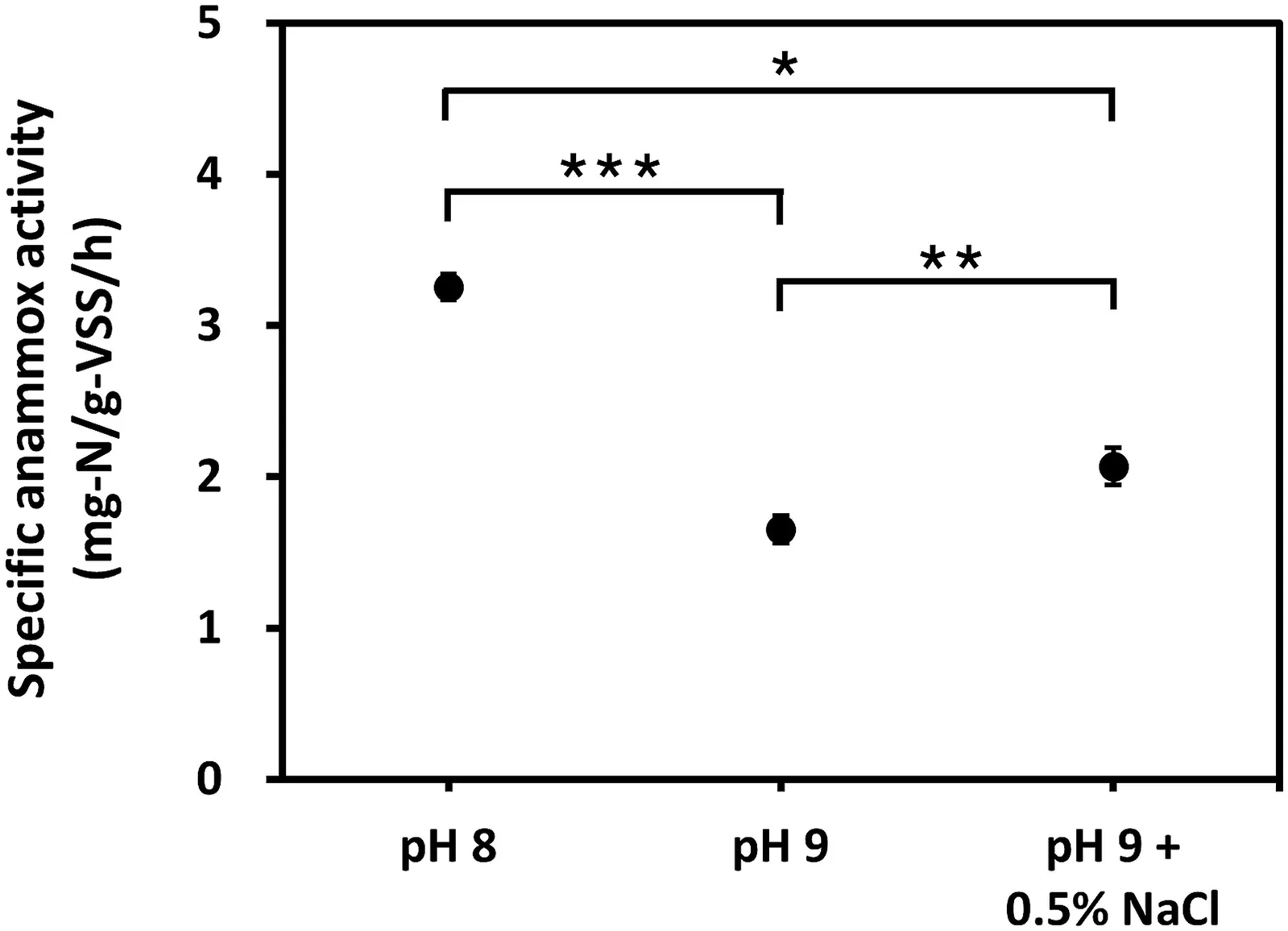
Sodium-stimulated recovery of specific anammox activity (SAA) under alkaline stress. SAA was calculated from the linear regression of the NH_3_ + NH_4_^+^ depletion profile (*R*^2^ > 0.97) and normalized to VSS. Statistical significance was determined using Student’s *t* test (^*^P < 0.05, ^**^  *P* < 0.01, ^***^P < 0.001). Error bars represent the standard deviation from three biological replicates.

To validate the genomic prediction of Mrp-mediated pH homeostasis, we assessed whether cation availability could mitigate alkaline inhibition. Because *Ca.* L. alkalitolerans was enriched in the NaCl-amended KJ reactor, sodium was selected as the test cation. Using pH 8 as the optimal baseline, batch assays were conducted at pH 9 as the alkaline control, a condition demonstrated to reduce activity by ~50% (Fig. 2A). Sodium effects were then tested by comparing pH 9 assays with or without 0.5% NaCl, a concentration demonstrated to be noninhibitory (*P* = 0.29; Fig. 2B). Consistent with the genomic prediction, the addition of 0.5% NaCl significantly mitigated alkaline inhibition, increasing SAA by 25.5% relative to the alkaline control (*P* < 0.01; Fig. 6).

### Metatranscriptomic profiles under alkaline and Na^+^-supplemented conditions

**Figure 7:**
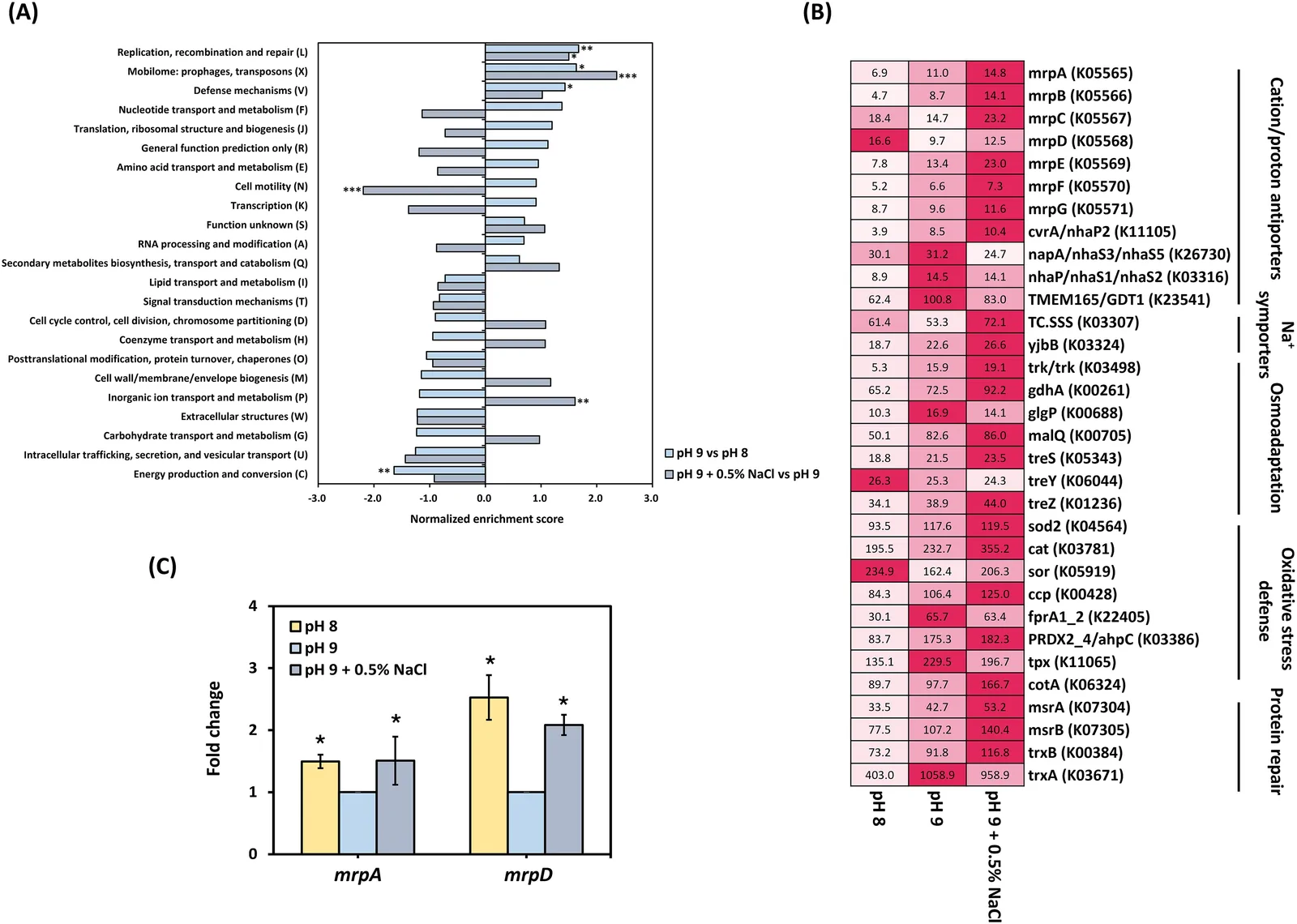
Transcriptional responses of *Ca*. Loosdrechtia alkalitolerans under alkaline (pH 9) and saline-alkaline (pH 9 + 0.5% NaCl) conditions. (A) Gene set enrichment analysis of COG functional categories. Adjusted *P* values were calculated using the Benjamini–Hochberg method (^*^P < 0.05, ^**^  *P* < 0.01, ^***^P < 0.001). (B) Mean transcripts per million (TPM) values of genes associated with ion and pH homeostasis, osmoadaptation, oxidative stress defense, and protein repair. Color intensity was scaled row-wise across the three conditions for each gene, with darker shading indicating higher TPM values. KEGG Orthology (KO) numbers are shown in parentheses; detailed gene definitions are provided in Supplementary Data Sheet 2. (C) Relative expression of *mrpA* and *mrpD* in *Ca*. Loosdrechtia alkalitolerans. Transcript levels were quantified by RT-qPCR using the 2^-∆∆Ct^ method, with the 16S rRNA gene of *Ca*. Loosdrechtia alkalitolerans as the internal control and the average expression at pH 9 set to 1. Statistical significance was determined using Student’s *t* test (^*^P < 0.05). Error bars represent the standard deviation from three biological replicates.

Metatranscriptomic analysis was conducted to investigate the gene expression profiles of *Ca.* L. alkalitolerans under saline-alkaline conditions using biomass from the batch assays (Fig. 6). COG-category-based GSEA indicated that alkaline stress significantly enriched genes associated with replication, recombination and repair (L), mobilome (X), and defense mechanisms (V), whereas energy production and conversion (C) was negatively enriched. Compared to alkaline stress alone, Na^+^ addition further enriched the replication/repair (L), mobilome (X), and inorganic ion transport and metabolism (P) categories, whereas cell motility (N) was negatively enriched (adjusted *P* < 0.05; Fig. 7A). The enrichment of the mobilome category was mainly attributed to putative transposases and insertion sequence (IS)-related genes, with a more pronounced enrichment after Na^+^ addition (Supplementary Data Sheet 3). Such stress-responsive upregulation of DNA mobility and genome-rearrangement functions may reflect enhanced genomic flexibility in *Ca.* L. alkalitolerans. Furthermore, consistent with the gene enrichment patterns, genes involved in ion and pH homeostasis, stress defense, and repair systems generally showed higher transcript abundances under alkaline and saline-alkaline conditions (Fig. 7B). In particular, the thioredoxin system genes (*trxA* and *trxB*) were highly expressed. This system repairs oxidized proteins and maintains cellular redox balance through disulfide-bond reduction coupled to NADPH oxidation [86]. Regarding ion and pH homeostasis, Mrp showed the clearest response to Na^+^ addition among the cation/proton antiporters identified in *Ca.* L. alkalitolerans. Most Mrp subunits had higher TPM values than in the alkaline control, although the differences were not statistically significant. To further verify *mrp* expression, RT-qPCR was performed on the core subunit genes *mrpA* and *mrpD* [45] using the 16S rRNA gene as the reference. Although alkaline stress alone downregulated the expression of both genes relative to the optimal pH 8 condition, Na^+^ supplementation reversed this effect, significantly increasing their expression compared with the alkaline control (*P* < 0.05; Fig. 7C). Together, these transcriptomic responses suggest that *Ca.* L. alkalitolerans copes with saline-alkaline stress by enhancing stress-defense and repair systems while increasing the potential for genomic flexibility. In parallel, the Na^+^-responsive activation of the Mrp antiporter may reinforce ion and proton homeostasis, collectively supporting its persistence in the saline-alkaline KJ reactor.

## Discussion

The taxonomic assignment of *Ca.* L. alkalitolerans reveals a discrepancy between phylogenomic topology and standard sequence identity thresholds. *Ca.* L. alkalitolerans shared higher full-length 16S rRNA gene identity with *Ca*. Jettenia spp. than with *Ca.* L. aerotolerans (Fig. S6), with all values falling below the proposed 94.5% genus delineation threshold [87]. Although this low identity suggests *Ca.* L. alkalitolerans might represent a novel genus, this interpretation was not supported by protein-based OGRIs or phylogenomic analysis. Given the reported limitations of AAI in classifying anammox lineages [6], we focused on the more robust phylogenomic evidence based on concatenated single-copy marker genes, which supports the monophyly of the *Ca*. Loosdrechtia clade with high bootstrap support (Fig. 3). On this basis, we defined *Ca.* L. alkalitolerans as a distinct species within the genus *Ca*. Loosdrechtia.

Saline-alkaline stress appears to define the realized niche of *Ca.* L. alkalitolerans, distinguishing it from other well-characterized anammox genera. In our reactors, glutamate supplementation selectively enriched *Ca*. Brocadia, consistent with its competitiveness in organic substrates-rich environments [88, 89]. By contrast, *Ca.* L. alkalitolerans did not respond to glutamate supplementation. Under salinity stress, *Ca.* K. stuttgartiensis maintained dominance, consistent with its documented tolerance of up to 6% salinity [13]. Conversely, *Ca.* L. alkalitolerans showed optimal activity at 0%–0.5% NaCl but was markedly inhibited at higher salinity (Fig. 2B). This tolerance exceeds that of nonhalophilic genera such as *Ca*. Brocadia and *Ca*. Jettenia but is lower than that of *Ca*. Kuenenia (Table S5). This pattern is in line with our phylogenomic analysis and the proposed evolutionary trajectory of anammox bacteria shaped by habitat, in which highly salinity-tolerant marine lineages emerged first and later freshwater lineages gradually reduced this trait [13]. Accordingly, *Ca.* L. alkalitolerans may occupy an intermediate evolutionary position between *Ca*. Kuenenia and *Ca*. Jettenia. In the KJ reactor, where mild salinity (0.5%) coincided with alkaline pH (~9.13; significantly higher than that in the FGA and SGA reactors, *P* < 0.001; Table S1), *Ca.* L. alkalitolerans outcompeted the halotolerant *Ca*. Kuenenia. This finding suggests that *Ca*. Kuenenia dominates under osmotic stress alone, whereas *Ca.* L. alkalitolerans gains a selective advantage when osmotic stress and alkaline stress co-occur. Competitive exclusion likely reflects superior pH and ion homeostasis, indicating specialization for saline-alkaline environments rather than general halophily. Because other anammox species persisted at low abundance in the sludge, future enrichment should create combined alkaline (pH 9–10) and saline (0.5%–1.0%) conditions to suppress co-occurring anammox species, thereby facilitating the purification of *Ca.* L. alkalitolerans for in-depth physiological characterization.

The Mrp antiporter complex likely represents a key mechanism that enables *Ca*. L. alkalitolerans to maintain intracellular homeostasis under saline-alkaline conditions. Neutrophilic bacteria grow over a broad external pH range (~5.5–9.0) while maintaining a narrow cytoplasmic pH range (~7.5–7.7) [80, 90]. Similarly, anammox bacteria such as *Ca.* K. stuttgartiensis tightly regulate riboplasmic pH at ~ 7.3 (pH 6.3 in the anammoxosome) across an external pH range of 6.5–7.8 [91]. Maintaining a stable intracellular pH is important for limiting intracellular free ammonia accumulation and buffering alkalinity generated during anammox metabolism. However, under more alkaline conditions, cells must actively import protons against a concentration gradient, driven by transmembrane electrical potential and mediated by cation/proton antiporters [80, 81]. Our genomic analysis suggested that in addition to encoding the basal cation/proton transport systems, *Ca.* L. alkalitolerans uniquely encodes a complete Mrp operon that is absent in other freshwater *Ca*. Brocadiaceae genomes. We then experimentally showed that the addition of 0.5% NaCl upregulated *mrp* expression and recovered anammox activity (Figs. 6 and 7). This response parallels the Na^+^-dependent pH homeostasis observed in *Halomonas* sp. Y2 [78]. The cation-dependent effect was further supported by the observation that K^+^ supplementation could similarly mitigate alkaline inhibition, whereas Li^+^ showed no beneficial effect (Fig. S10). Although direct biochemical validation of the Mrp complex and the precise mechanisms underlying the differential cation effects remain to be elucidated, the unique genomic presence of this complex, combined with its specific transcriptional upregulation and the cation-dependent recovery of anammox activity, suggests that Mrp-mediated pH homeostasis is a critical adaptive trait for *Ca.* L. alkalitolerans under alkaline stress. Other alkaline tolerance strategies, such as regulating acid-producing metabolic pathways [80], should also be considered. Carbonic anhydrase, which supports intracellular inorganic carbon balance [92], may also be important for the growth of chemolithoautotrophic anammox bacteria under alkaline conditions. However, based on current genome annotations, no clear evidence for acid-producing metabolic pathways or carbonic anhydrase genes was identified in the *Ca.* L. alkalitolerans genome. These unresolved mechanisms warrant further investigation.

In addition to pH homeostasis, the metatranscriptomic results suggest that *Ca.* L. alkalitolerans activates a broader stress-adaptive response under saline-alkaline conditions. The enrichment of genes associated with transposases and IS elements has rarely been described in anammox bacteria, suggesting that genomic plasticity may represent an under-recognized component of stress adaptation in this lineage. Elevated transposase expression has been linked to dynamic and stressful environments and may contribute to adaptive flexibility in such environments [93]. Although the evolutionary consequences of this response remain unclear, the coordinated activation of stress-defense, repair, and mobile-element-associated functions collectively supports the persistence of *Ca.* L. alkalitolerans under saline-alkaline stress and indicates broader ecological flexibility.

*Ca.* L. alkalitolerans appears to occupy a broad ecological niche that extends well beyond strictly haloalkaline environments. A database search identified 175 amplicon datasets that contained sequences closely related to this species, primarily from freshwater, soil, and activated sludge ecosystems (Fig. S11A). Approximately 60% of these records overlapped with *Ca.* L. aerotolerans, indicating a shared core niche within the genus; the remaining nonoverlapping records suggested lineage-specific diversification (Fig. S11B). This broad environmental distribution indicates that the saline-alkaline tolerance observed in our enrichment assays represents latent adaptive potential that allows the species to persist under chemically fluctuating conditions rather than restricting it to extreme habitats. Furthermore, three MAGs closely related to *Ca.* L. alkalitolerans were recovered from wastewater treatment systems (WH-1, HSAMX1, and AMX2) [94, 95]. Sequences highly similar (i.e. ≥97% 16S rRNA gene identity) to *Ca.* L. alkalitolerans have not been detected in open ocean environments (seawater or sediments). This ecological distribution is consistent with the inability of *Ca.* L. alkalitolerans to sustain anammox activity under marine salinity conditions (i.e. 3.5% NaCl; Fig. 2B), supporting its freshwater-derived origin and suggesting its potential application in engineered systems.

In summary, the successful enrichment of *Ca.* L. alkalitolerans marks a crucial advancement in the physiological characterization of the anammox guild. Before this study, the genus *Ca*. Loosdrechtia had not been studied with a highly enriched culture. The present study established it as an experimentally tractable model alongside *Ca*. Brocadia, *Ca*. Kuenenia, *Ca*. Jettenia, and *Ca*. Scalindua. As the anammox bacterium enriched under saline-alkaline stress conditions, *Ca.* L. alkalitolerans offers a unique platform for investigating extremophilic adaptations within the *Planctomycetes* phylum. The high-quality genomic and physiological data generated in our study provide a reference framework for the *Ca*. Loosdrechtia clade, improving taxonomic resolution in future environmental surveys and expanding our understanding of anammox bacterial ecology.

### Etymology description of *Ca.* Loosdrechtia alkalitolerans

Alkalitolerans (al.ka.li.to.le’rans. N.L. neut. adj., derived from *alkali* and the Latin participle *tolerans*, tolerant) refers to the organism’s ability to tolerate alkaline conditions. The reddish brown appearance of sludge dominated by *Ca.* L. alkalitolerans and corresponding fluorescence in situ hybridization images are presented in Fig. S12. Synonym: *Ca*. Loosdrechtia sp. KJ [30].

## Supplementary Material

Web_Material_wrag221

## Data Availability

All sequencing data generated in this study have been deposited in the NCBI database under BioProject accession number PRJNA1313157. The whole-genome sequencing project for *Ca*. Loosdrechtia alkalitolerans has been deposited in GenBank under accession number JBQWCT000000000.
