# Supplementary material for: Enrichment and characterization of a haloalkaline-tolerant anammox bacterium
Source: ISME J. 2026 Aug 27;20(1):wrag221. doi: 10.1093/ismejo/wrag221 (PMC13597103; doi:10.1093/ismejo/wrag221)
Supplement: Supplementary file 1 [file web_material_wrag221.zip › Final_SI_KJ_pH_wrag221.pdf]

Supplementary Information

Enrichment and Characterization of a Haloalkaline-tolerant Anammox Bacterium

Han-Yin Chuang[1], Jer-Horng Wu[1]*, Shan-Hua Chen[1], Wei-Yu Chen[2], Li-Jin Fong[1] and Yi-Ting Lin[1]

[1]Department of Environmental Engineering, National Cheng Kung University, No.1, University Road, East District, Tainan City 70101, Taiwan

[2]Department of Environmental Engineering, National Ilan University, No.1, Sec. 1, Shennong Rd., Yilan City, Yilan County 26047, Taiwan

Running title: Haloalkaline-tolerant Anammox Bacterium

*Corresponding author: Jer-Horng Wu

Mail Address: Department of Environmental Engineering, National Cheng Kung University, No.1, University Road, East District, Tainan City 70101, Taiwan

Email: enewujh@ncku.edu.tw

This manuscript contains 4 supplementary texts, 6 supplementary tables, 12 supplementary figures, and 3 supplementary data sheets.

Text S1. Chemical analyses and the evaluation of nitrogen removal performance.
Text S2. RT-qPCR analysis of *mrp* gene expression.
Text S3. Data processing of 16S rRNA gene amplicon sequencing data.
Text S4. Global occurrence of *Ca*. Loosdrechtia alkalitolerans.
Table S1. Summary of operational parameters and performance of anammox reactors.
Table S2. Composition of the synthetic influent.
Table S3. Primer sets designed for *Ca*. Loosdrechtia alkalitolerans.
Table S4. Genomic features of the anammox bacterial MAGs retrieved from the NCBI database.
Table S5. Summary of pH and salinity tolerance in *Ca.* Loosdrechtia alkalitolerans and other representative anammox species from different genera.
Table S6. Genomic features of MAGs with relative abundance >1% in the KJ reactor**.**
Figure S1. Performance of the KJ reactor operated without NaCl prior to the batch assays.
Figure S2. Bacterial community composition of sludge from the KJ reactor before batch assays.
Figure S3. Performance of FGA (A, B), SGA (C, D), and KJ (E, F) reactors.
Figure S4. (A) Sequencing batch reactor (SBR) operation mode of the KJ reactor and changes in (B) pH, (C) free ammonia, and (D) total ammonia nitrogen over a single SBR cycle.
Figure S5. Phylogenetic placement of anammox bacteria.
Figure S6. Pairwise identity matrix of full-length 16S rRNA genes.
Figure S7. Pairwise orthologous average nucleotide identity (OrthoANI) matrix.
Figure S8. Pairwise average amino acid identity (AAI) matrix.
Figure S9. Phylogenetic analysis of Mrp complex subunits.
Figure S10. Cation-specific recovery of specific anammox activity under alkaline stress.
Figure S11. Global occurrence and environmental overlap of *Ca*. Loosdrechtia species.
Figure S12. *Ca.* Loosdrechtia alkalitolerans-dominant biomass enriched in this study.
Supplementary Data Sheet 1. Sequence accession numbers included in the custom database.
Supplementary Data Sheet 2. Functional annotation of the *Ca*. Loosdrechtia alkalitolerans (JSbin17) MAG reconstructed in this study.
Supplementary Data Sheet 3. Core-enrichment mobilome ORFs assigned to COG category X identified by GSEA.

Text S1. Chemical analyses and the evaluation of nitrogen removal performance.

The supernatants from the reactors and batch assays were first filtered through a 0.2 μm nylon membrane before analysis. NH_4^+ and NO_2^- were determined colorimetrically using the Nessler reagent set (HACH, USA) and the naphthylethylenediamine method (APHA 4500- NO_2^- B [1]), respectively. NO_3^- was quantified by ion chromatography (Dionex ICS-1100 ion chromatography, Thermo Fisher Scientific, USA) equipped with a Dionex IonPac AS9-HC column. pH was measured by a benchtop pH meter (SUNTEX, Taiwan).

Total nitrogen loading rate (TNLR), total nitrogen removal rate (TNRR), and total nitrogen removal efficiency (TNRE) were calculated according to equations (1)-(4). Where $NH_4^+{}_{inf}$ and $NO_2^-{}_{inf}$ represent ammonium and nitrite concentrations (mg-N/L) in the influent, and $NH_4^+{}_{eff}$, $NO_2^-{}_{eff}$, and $NO_3^-{}_{eff}$ represent ammonium, nitrite, and nitrate concentrations (mg-N/L) in the effluent. HRT denotes the hydraulic retention time (day) of reactors.

$$\Delta N\ (\text{mg-N/L}) = (NH_4^+{}_{inf} + NO_2^-{}_{inf}) - (NH_4^+{}_{eff} + NO_2^-{}_{eff} + NO_3^-{}_{eff}) \qquad \text{eq (1)}$$

$$\text{TNLR (kg-N/m}^3\text{/day)} = \frac{NH_4^+{}_{inf} + NO_2^-{}_{inf}\ (\text{kg-N/m}^3)}{\text{HRT (day)}} \qquad \text{eq (2)}$$

$$\text{TNRR (kg-N/m}^3\text{/day)} = \frac{\Delta N\ (\text{kg-N/m}^3)}{\text{HRT (day)}} \qquad \text{eq (3)}$$

$$\text{TNRE (\%)} = \frac{\Delta N\ (\text{mg-N/L})}{NH_4^+{}_{inf} + NO_2^-{}_{inf}\ (\text{mg-N/L})} \times 100\% \qquad \text{eq (4)}$$

Text S2. RT-qPCR analysis of *mrp* gene expression.

RNA was extracted using the RNeasy PowerWater kit (Qiagen, Germany). To eliminate potential genomic DNA carryover, RNA aliquots were treated with RQ1 RNase-Free DNase (Promega, USA). First-strand complementary DNA (cDNA) was then synthesized from the DNase-treated RNA using random primers and a GScript First-Strand Synthesis kit (GeneDireX, USA). All procedures were performed according to the respective manufacturers' instructions. Concentrations and the quality of RNA and cDNA were assessed on a NanoDrop One microvolume UV-Vis spectrophotometer (Thermo Fisher Scientific, USA).

Oligonucleotide primers targeting the 16S rRNA gene, *mrpA*, and *mrpD* specific to *Ca*. Loosdrechtia alkalitolerans were designed using NCBI Primer-BLAST [2] on the basis of the *Ca*. L. alkalitolerans genome [3]. Primer pairs (Table S3) were selected according to the following criteria: (i) primer length of 17-25 bp; (ii) amplicon length of 100-250 bp; (iii) GC content of 40-60%; (iv) melting temperature (T_m) of 55-65 °C

with a difference of < 2 °C between reverse and forward primers; and (v) self-complementarity score <5 and 3' self-complementarity score <3. Primer pair specificity was assessed by BLAST searches against the NCBI nt database with default parameters. The primer set targeting the 16S rRNA gene was further validated with SILVA TestProbe3.0 to examine its taxonomic coverage and potential off-target matches.

qPCR reactions were performed on a CFX Duet Real-Time PCR System (Bio-Rad, USA) using SYBR Green Premix Ex Taq (TaKaRa Bio, Japan). All reactions were run with the same thermal cycling program: denaturation at 95 °C for 3 min, followed by 40 cycles of 95 °C for 10 s, 60 °C for 45 s, and 72 °C for 30 s. A melting curve analysis (65-95 °C, 0.5 °C/s) and agarose gel electrophoresis were subsequently performed to verify the specificity of the amplified products. Amplification efficiencies for all primer sets were within ±10% (92.6%-101.6%). Relative expression ratios of *mrpA* and *mrpD* were calculated using $2^{-\Delta\Delta Ct}$ method, with the 16S rRNA gene of *Ca*. L. alkalitolerans used as the internal control. All reactions were performed in biological and technical triplicate.

Text S3. Data processing of 16S rRNA gene amplicon sequencing data.

The obtained sequencing reads were processed in Qiime2 (v2024.10) [4]. Raw reads were first demultiplexed, quality filtered, and merged into pair-end reads, followed by denoising with the q2-dada2 plugin [5] to remove chimeras and infer amplicon sequence variants (ASVs). Representative sequences for each ASV were taxonomically classified using a scikit-learn Naive Bayesian classifier trained on the SILVA 138.2 SSURef_NR99 rRNA database using the q2-feature-classifier plugin with default settings [6].

Text S4. Global occurrence of *Ca.* Loosdrechtia alkalitolerans.

The full-length 16S rRNA gene sequence of *Ca.* Loosdrechtia alkalitolerans (1592 bp) was used as a query in IMNGS [7] with a 95% similarity threshold and a minimum sequence length of 200 bp. Although a 94.5% 16S rRNA gene identity has been proposed as the genus-level threshold [8], *Ca.* Loosdrechtia aerotolerans shares only 91.6% identity with *Ca.* Loosdrechtia alkalitolerans (Figure S6) and would therefore not be retrieved in this analysis. All retrieved sequences were subsequently subjected to *blastn* against the 16S rRNA gene of *Ca.* Loosdrechtia alkalitolerans to validate the results, and only datasets with more than five hits were retained. The corresponding metadata of these datasets were then collected to summarize the geographic distribution of *Ca.* Loosdrechtia alkalitolerans-affiliated bacteria, and the results were visualized in R v4.4.1 using the packages ggplot2 and maps.

Table S1. **Summary of operational parameters and performance of the FGA, SGA, and KJ reactors.**

Parameter	FGA	SGA							KJ		
Operational day	1-310	1-27	28-56	57-116	117-170	171-197	198-266	267-310	1-288	289-613	614-738
Add NaCl (%, w/v)	0	0.5	1.5	2.5	3.5	2.5	1.5	0.5	0.5	0.5	0
Influent glutamate (mM)	0.2	0.2	0.2	0.2	0.2 → 1	1	1	1	0	0	0
Influent NH_4^+ (mg-N/L)	192 ± 13	234 ± 19	176 ± 24	196 ± 10	202 ± 6	202 ± 6	198 ± 4	196 ± 10	200[a]	295 ± 15	293 ± 11
Influent NO_2^- (mg-N/L)	207 ± 16	182 ± 17	187 ± 27	208 ± 12	216 ± 18	217 ± 4	208 ± 5	205 ± 10	200[a]	333 ± 14	332 ± 12
Effluent pH	7.98 ± 0.44	8.33 ± 0.32	8.09 ± 0.29	8.33 ± 0.24	8.44 ± 0.20	8.41 ± 0.15	7.95 ± 0.31	7.76 ± 0.34	NM[b]	9.13 ± 0.25	8.52 ± 0.14
HRT (day)	1.5	1.5	1.5	1.5	1.5	1.5	1.5	1.5	1.5	1.5 → 0.7	0.7
TNLR[c] (kg-N/m^3/day)	0.27 ± 0.02	0.28 ± 0.01	0.24 ± 0.03	0.27 ± 0.01	0.28 ± 0.01	0.28 ± 0.01	0.27 ± 0.01	0.27 ± 0.01	0.27[a]	0.43 → 0.94	0.94 ± 0.02
TNRE[d] (%)	88.3 ± 2.7	90.4 ± 4.3	88.1 ± 1.2	69.2 ± 14.7	17.7 ± 11.4	11.8 ± 5.4	17.4 ± 6.5	74.7 ± 12.1	NM[b]	82.5 ± 1.9	84.8 ± 3.6

[a] Designed concentration, not experimentally measured; [b] Not measured; [c] Total nitrogen loading rate; [d] Total nitrogen removal efficiency.

Table S2. Composition of the synthetic influent.

Constituents	Concentration
NH_4Cl (mg-N/L)	Adjusted according to experimental design
$NaNO_2$ (mg-N/L)	Adjusted according to experimental design
NaCl (g/L)	Adjusted according to experimental design
Monosodium L-glutamate (mM)	Adjusted according to experimental design
Other nutrients (mg/L)	
$KHCO_3$	500
KH_2PO_4	50
KCl	50
$MgSO_4 \cdot 7H_2O$	25
$CaCl_2 \cdot 2H_2O$	100
NaOH	0.5
$Na_2SeO_3 \cdot 5H_2O$	0.003
Na_2WO_4	0.005
$MnSO_4 \cdot H_2O$	0.034
H_3BO_3	0.05
$ZnCl_2$	0.07
$Na_2MoO_4 \cdot H_2O$	0.073
$CuCl_2 \cdot 2H_2O$	0.02
$NiCl_2 \cdot 6H_2O$	0.024
$CoCl_2 \cdot 6H_2O$	0.08
$FeSO_4 \cdot 7H_2O$	1

Table S3. Primer sets designed for *Ca*. Loosdrechtia alkalitolerans.

Target gene	Primer	Sequence (5’ → 3’)	Primer length (bp)	Product length (bp)	Tm (℃)	GC content (%)
16S rRNA	KJ_16S_482F	GGA GTT AAG AAA GGC GGG AGA	21	220	59.45	52.38
	KJ _16S_701R	GCA CTC TAG CCC TGT AGT ATC AC	23		60.00	52.17
mrpA	KJ_mrpA_483F	AGA GGC TGT AAA TGC CGG AC	20	124	60.11	55.00
	KJ_mrpA_606R	AAT ACC ACC CGG ACG GAA AC	20		60.03	55.00
mrpD	KJ_mrpD_871F	GCT GGT TCG GTA ATG GCT CT	20	250	60.11	55.00
	KJ_mrpD_1120R	TGG GCA TTC TGG TAG CAA GG	20		60.04	55.00

Table S4. Genomic features of the anammox bacterial MAGs retrieved from the NCBI genome database in August 2025.

#	Anammox bacterial species	# of contigs	Size (Mb)	Completeness (%)	Contamination (%)	Accession #
1	*Ca.* Brocadia fulgida RU1	315	3.5	96.2	2.7	GCA_000987375.1
2	*Ca.* Brocadia sp. AM9	1	4.0	100.0	2.7	GCA_021650915.1
3	*Ca.* Brocadia fulgida Bin_44	134	2.8	90.1	1.6	GCA_019186885.1
4	*Ca.* Brocadia sp. UTAMX2	148	3.4	100.0	1.6	GCA_002050315.1
5	*Ca.* Brocadia pituitae	1	4.1	98.9	2.7	GCA_017347445.1
6	*Ca.* Brocadia caroliniensis	209	3.7	93.3	2.7	GCA_002009475.1
7	*Ca.* Brocadia sapporoensis 40	132	2.9	92.2	0.5	GCA_001753675.2
8	*Ca.* Brocadia sapporoensis BROCD001	38	3.2	99.7	1.6	GCA_019455295.1
9	*Ca.* Brocadia sapporoensis STL-AMX2	2	3.5	100.0	1.6	GCA_046350575.1
10	*Ca.* Brocadia sp. Ega	1	3.3	97.5	1.6	GCA_016699285.1
11	*Ca.* Brocadia sp. UTAMX1	36	3.1	100.0	1.6	GCA_002050325.1
12	*Ca.* Brocadia sinica BroJett002	23	4.1	97.8	1.6	GCA_016860665.1
13	*Ca.* Brocadia sinica HBSIN01	89	4.0	97.8	1.6	GCA_022180465.1
14	*Ca.* Brocadia sinica JPN1	3	4.1	97.8	1.6	GCA_000949635.1
15	*Ca.* Brocadia sp. AMX2	88	3.9	97.8	1.6	GCF_030263755.1
16	*Ca.* Jettenia caeni	4	4.1	100.0	3.8	GCA_000296795.1
17	*Ca.* Jettenia caeni YC_1	1	4.0	100.0	3.8	GCA_030583625.1
18	*Ca.* Jettenia caeni YC_1 JETCAE04	95	3.9	100.0	3.3	GCA_022180965.1
19	*Ca.* Jettenia ecosi J2	223	3.9	100.0	3.4	GCA_005524015.1
20	*Ca.* Jettenia sp. AM49	1	3.8	95.6	1.6	GCA_021650895.1

21	*Ca.* Jettenia sp. CY_1	1	3.8	100.0	1.6	GCA_030583645.1
22	*Ca.* Loosdrechtia aerotolerans	61	3.3	96.7	3.3	GCA_021646405.1
23	*Ca.* Loosdrechtia sp. HSAMX1	108	3.3	98.9	1.6	GCA_049311485.1
24	*Ca.* Brocadiaceae bacterium WH-1	1	3.5	98.9	3.8	GCA_030583665.1
25	*Ca.* Planctomycetales bacterium isolate AMX2	102	3.5	98.9	2.7	GCA_900696655.1
26	*Ca.* Kuenenia stuttgartiensis CSTR1	1	4.3	97.8	1.6	GCA_011066545.1
27	*Ca.* Kuenenia stuttgartiensis HKUEN01	391	4.2	96.4	9.3	GCA_022180985.1
28	*Ca.* Kuenenia stuttgartiensis MBR1	1	4.4	97.8	1.6	GCA_900232105.1
29	*Ca.* Kuenenia hertensis YC6	89	4.2	97.8	2.7	GCA_021646465.1
30	*Ca.* Scalindua brodae RU1	282	4.1	92.7	2.3	GCA_000786775.1
31	*Ca.* Scalindua japonica husup_a2	47	4.8	95.5	3.4	GCA_002443295.1
32	*Ca.* Scalindua rubra Ru_enrich_A1	100	4.0	90.5	2.3	GCA_002632345.1
33	*Ca.* Scalindua sp. SCAELEC01	106	4.6	96.6	3.4	GCA_004282745.1
34	*Ca.* Scalindua sp. SCALA701	122	4.5	96.6	3.4	GCA_022181045.1
35	*Ca.* Scalindua sp. RBC_AMX2	2	4.3	97.7	4.5	GCA_031316235.1
36	*Ca.* Scalindua sp. AMX11	121	4.6	96.6	4.5	GCA_004351875.1

Table S5. Summary of pH and salinity tolerance in *Ca.* Loosdrechtia alkalitolerans and other representative anammox species from different genera.

	Ca. Loosdrechtia alkalitolerans	*Ca*. Kuenenia stuttgartiensis	*Ca*. Brocadia sinica	*Ca*. Jettenia caeni	*Ca*. Scalindua sp.
pH range with detectable activity	7.0-10.0 [a]	6.5-9.0	6.5-8.8	6.5-8.5	6-8.5
Optimal pH	8.0	8.0	8.0-8.2	8.0	7.5
Salinity range with detectable activity (%)	0-3.0	0-6.0	0-2.0	0-2.0	0.25-10.0
Optimal salinity (%)	0-0.5	0.25-1.5	0-0.25	0-0.25	1.5-3.0
Reference	This study	[9, 10]	[10, 11]	[10, 12]	[10, 13]

[a] pH values below 7.0 were not determined in this study.

Table S6. Genomic features of MAGs with relative abundance >1% in the KJ reactor.

Bin ID	GTDB Taxonomy	Comp.[a] (%)	Cont.[b] (%)	GC (%)	Genome size (bp)	# of contigs
JS_bin17	d__Bacteria;p__Planctomycetota;c__Brocadiia;o__Brocadiales;f__Brocadiaceae;g__Loosdrechtia;s__Loosdrechtia sp900696655	98.9	1.65	41.6	3394582	124
JS_bin29	d__Bacteria;p__Planctomycetota;c__Brocadiia;o__Brocadiales;f__Brocadiaceae;g__Kuenenia;s__Kuenenia stuttgartiensis_A	96.7	0.55	40.7	3689155	263
JS_bin2	d__Bacteria;p__Chloroflexota;c__Anaerolineae;o__Promineifilales;f__Promineifilaceae;g__CAADGU01;s__CAADGU01 sp01458457	98.2	4.73	56.5	7210611	138
JS_bin5	d__Bacteria;p__Pseudomonadota;c__Gammaproteobacteria;o__XN24;f__XN24	89.6	2.41	66.8	3447157	57
JS_bin13	d__Bacteria;p__Bacteroidota;c__Ignavibacteria;o__Ignavibacteriales;f__Melioribacteraceae;g__SURF-28	74.9	1.68	34.9	3165963	109
JS_bin24	d__Bacteria;p__Pseudomonadota;c__Gammaproteobacteria;o__Burkholderiales;f__Burkholderiaceae;g__Rubrivivax	90.1	1.56	72.1	3776981	102
JS_bin30	d__Bacteria;p__Patescibacteria;c__4484-211;o__JAGQKX01;f__JAGQKX01	74.4	3.37	47.1	987850	11
JS_bin34	d__Bacteria;p__Bacteroidota;c__Ignavibacteria;o__Ignavibacteriales;f__Melioribacteraceae;g__SURF-28;s__SURF-28 sp020724265	88.8	3.35	34.3	3660006	147
JS_bin35	d__Bacteria;p__Pseudomonadota;c__Gammaproteobacteria;o__Burkholderiales;f__Burkholderiaceae;g__Calidifontimicrobium	92.2	1.40	72.1	4228069	94
JS_bin41	d__Bacteria;p__Pseudomonadota;c__Gammaproteobacteria;o__GCA-2729495;f__GCA-2729495;g__JAFIFS01	89.2	1.73	67.1	2827725	55

[a] completeness; [b] contamination

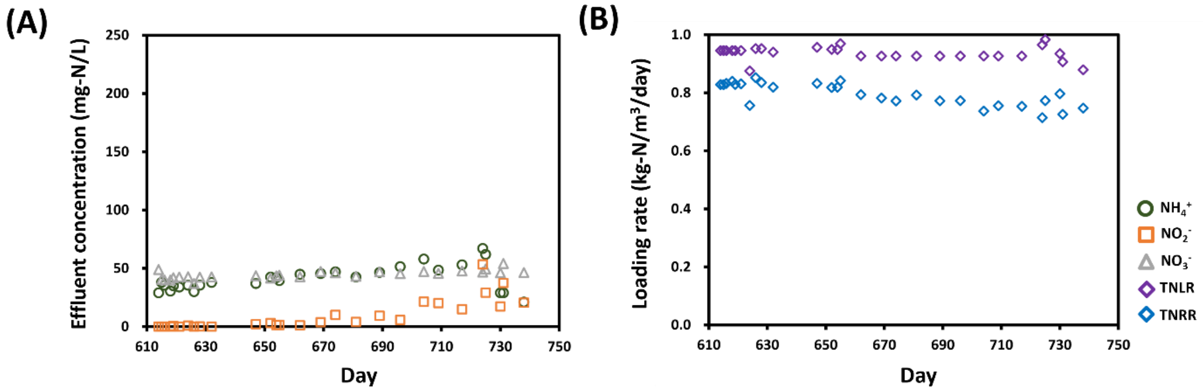

Figure S1. Performance of the KJ reactor (days 614-738) operated without NaCl prior to the batch assays. TNLR, total nitrogen loading rate; TNRR, total nitrogen removal rate.

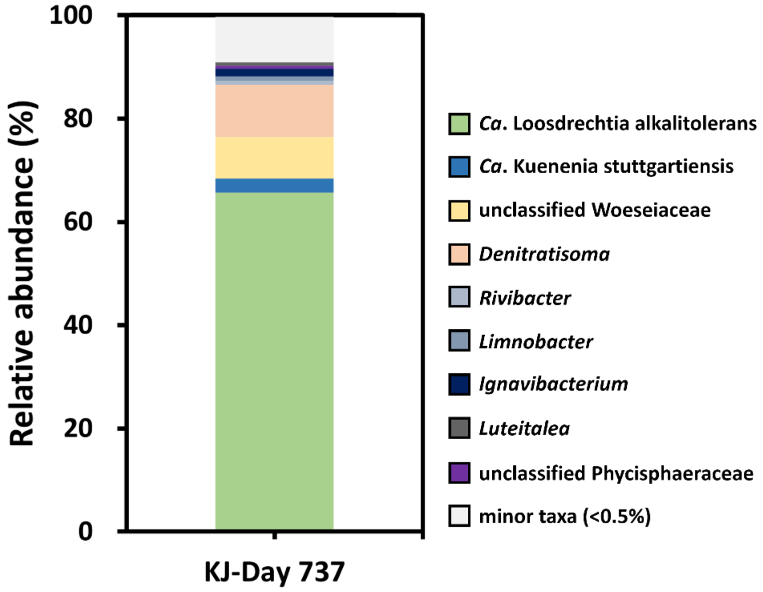

Figure S2. Bacterial community composition of sludge from the KJ reactor prior to the batch assays. Taxa with relative abundance <0.5% are grouped as minor taxa.

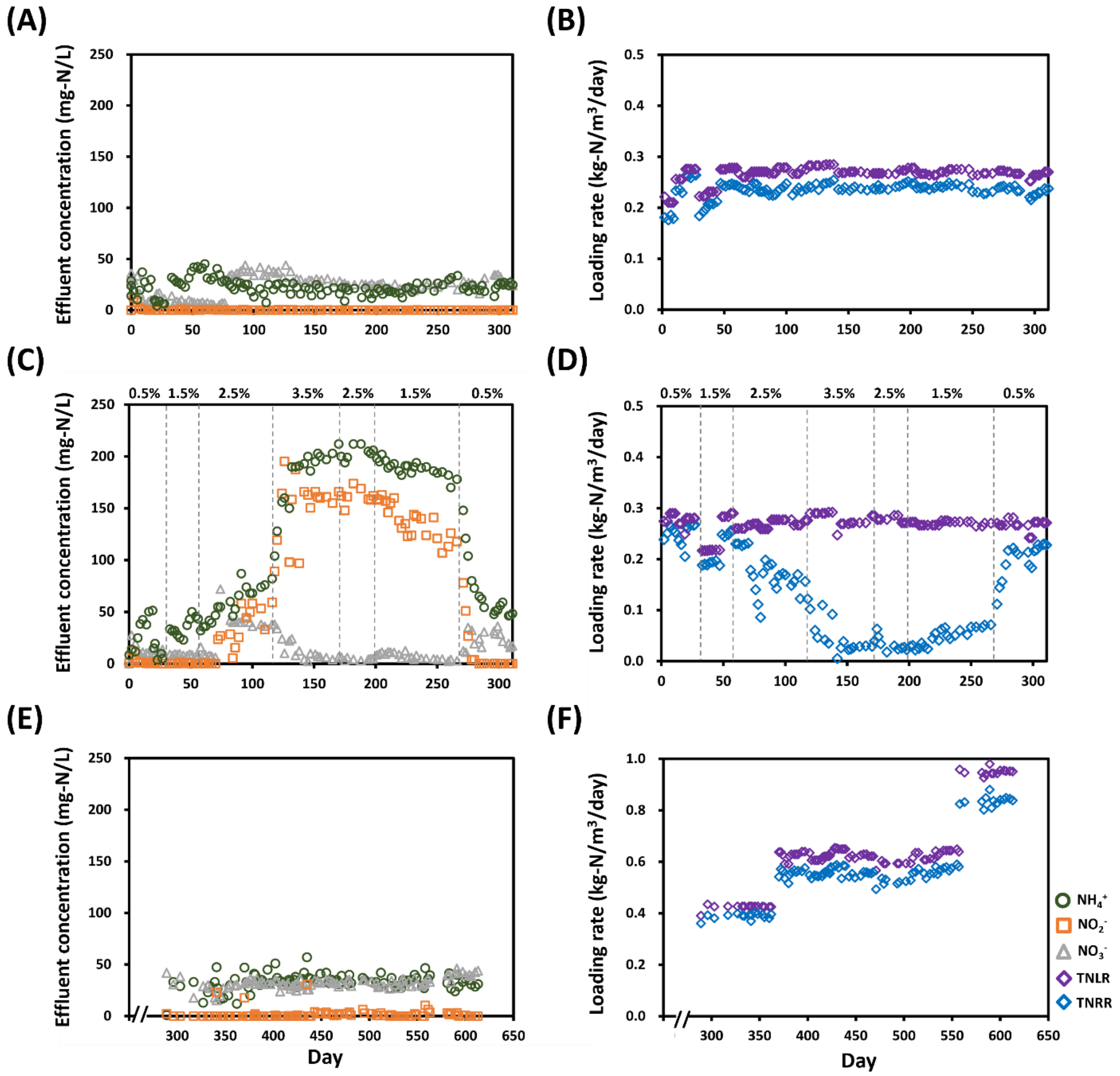

Figure S3. Performance of the FGA reactor (A, B), SGA reactor (C, D), and KJ reactor (E, F). Vertical dashed lines in **(C)** and **(D)** indicate phase boundaries defined by the salinity (added NaCl % (w/v) shown at the top of each panel). TNLR, total nitrogen loading rate; TNRR, total nitrogen removal rate.

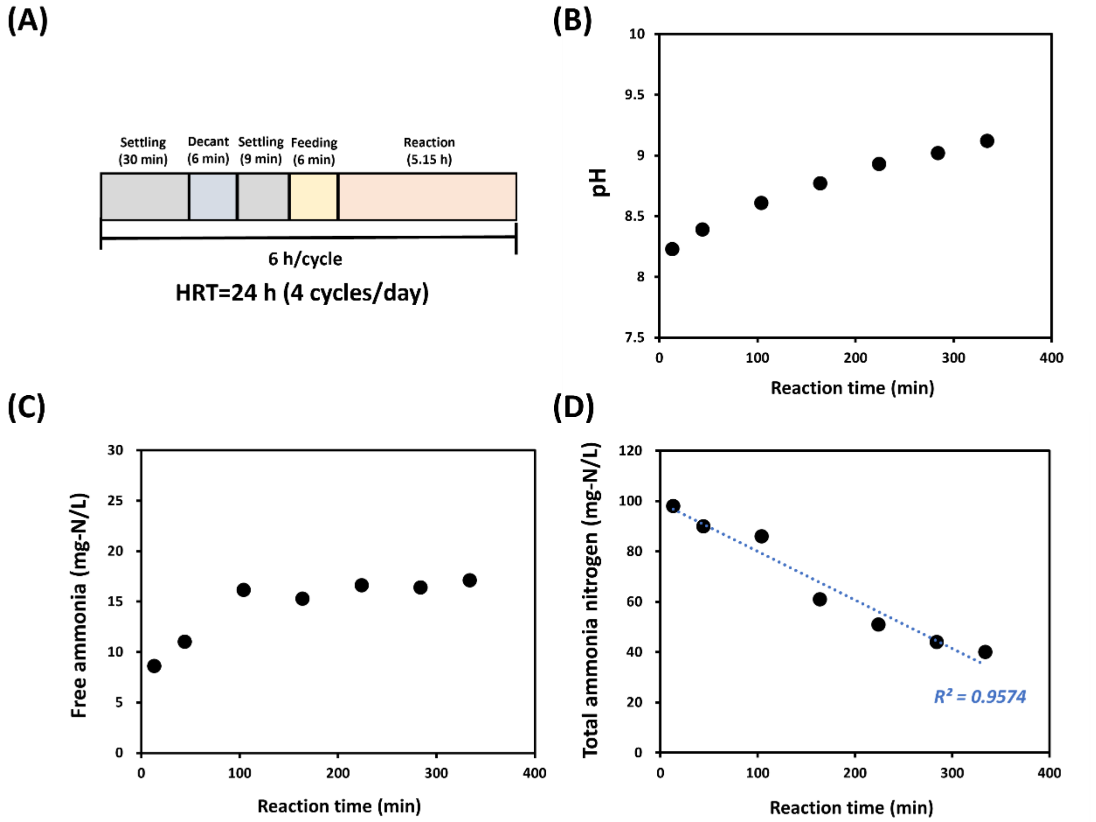

Figure S4. (A) Sequencing batch reactor (SBR) operation mode of the KJ reactor and changes in (B) pH, (C) free ammonia, and (D) total ammonia nitrogen over a single SBR cycle. Free ammonia concentrations were calculated according to Anthonisen et al. (1976) [14].

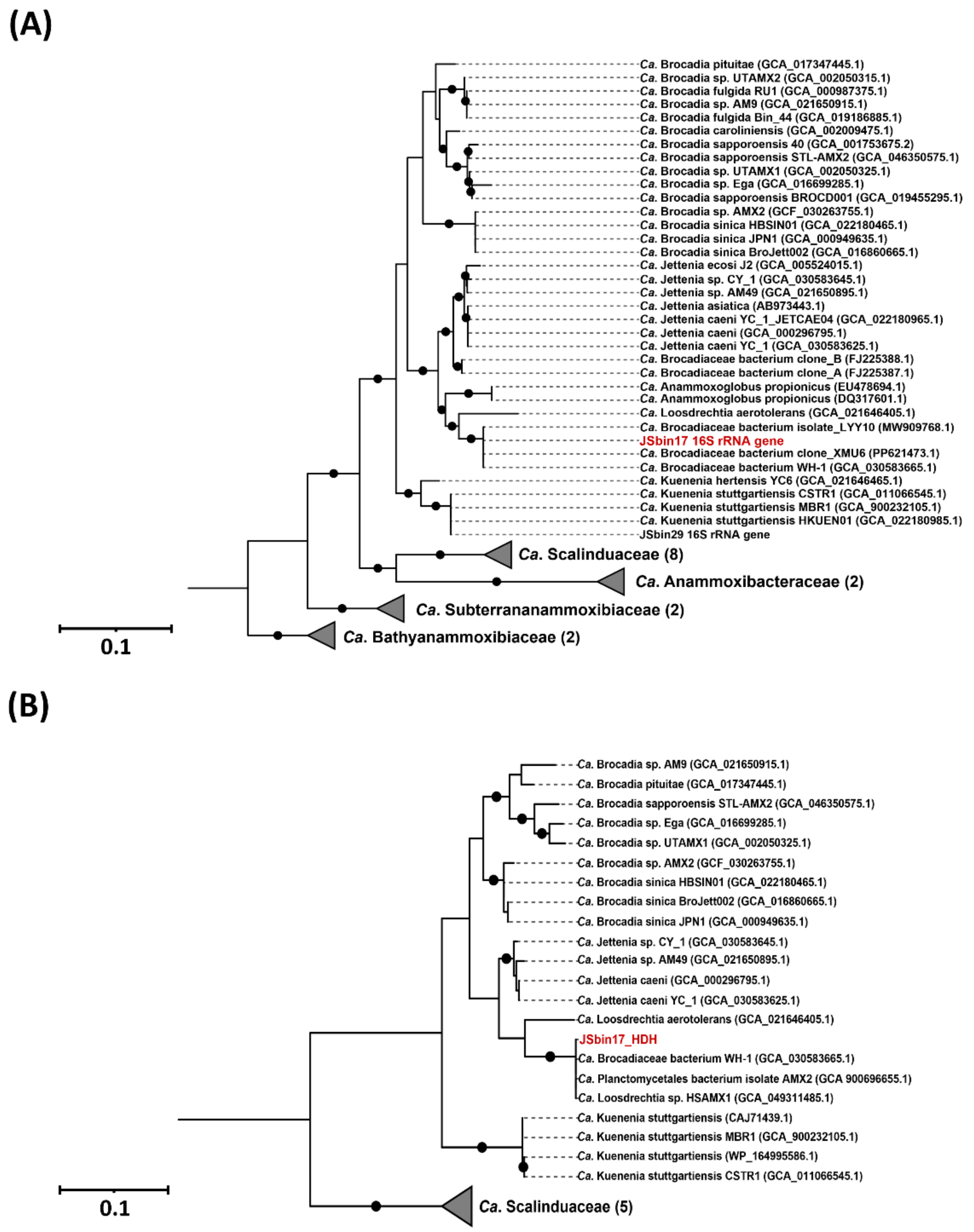

Figure S5. Phylogenetic placement of anammox bacteria. (A) Maximum-likelihood phylogenetic tree based on full-length 16S rRNA genes. Sequences retrieved from anammox bacterial MAGs were aligned with MUSCLE5, trimmed with trimAl (-automated1 option), and the tree was inferred with IQ-TREE3 under the best-fit model GTR+F+I+R2. **(B)** Maximum-likelihood phylogenetic tree based on hydrazine dehydrogenase (HDH) amino acid sequences. Sequences retrieved from anammox bacterial MAGs were aligned with MAFFT (L-INS-i option), trimmed with trimAl (-automated1 option), and the tree was inferred with IQ-TREE3 under the best-fit model WAG+R3. For both trees, model selection was performed using ModelFinder with 1,000 ultrafast bootstrap replicates; bootstrap values >90 are shown in solid circle. The scale bar shows the estimated number of substitutions per site. Deep-branching clades were

collapsed, with the number of sequences in each clade indicated in parentheses. Accession numbers for individual entries are also provided in parentheses. The *Ca.* Loosdrechtia alkalitolerans (MAG JSbin17) sequence is highlighted in red.

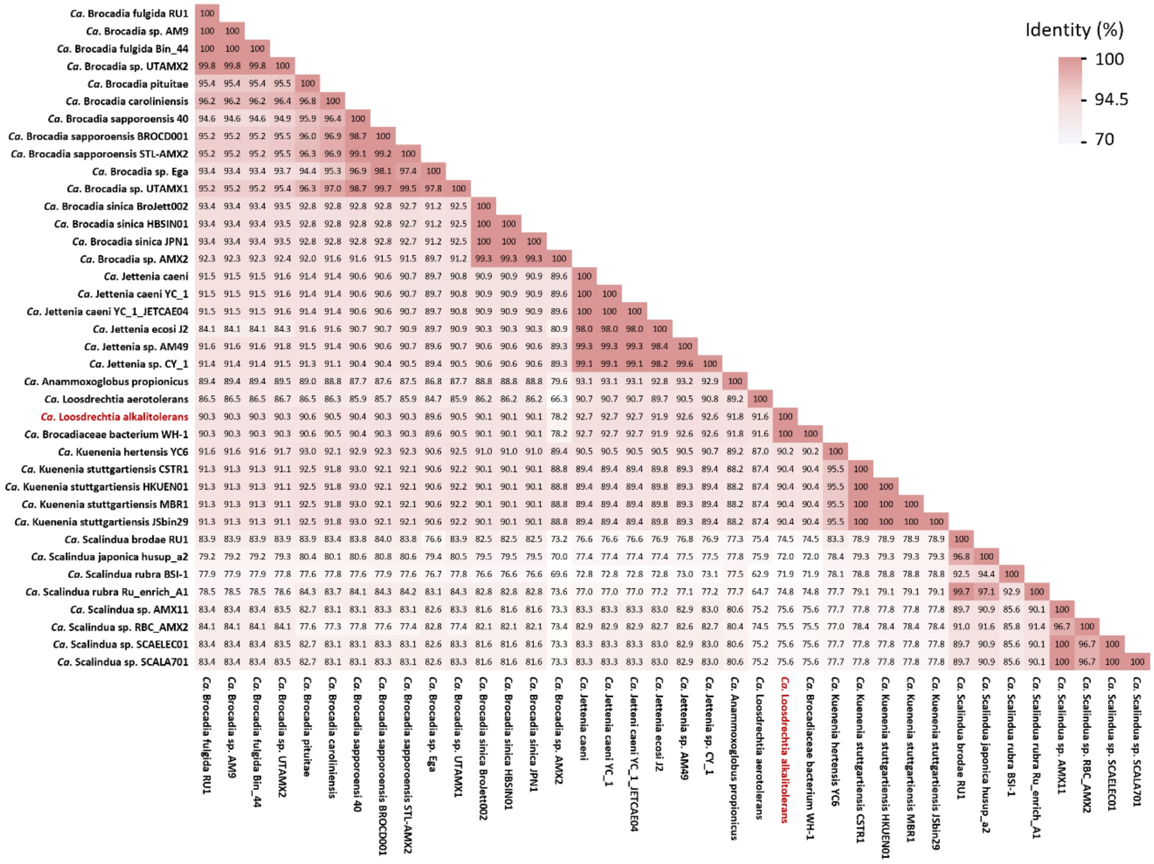

Figure S6. Pairwise identity matrix of full-length 16S rRNA gene sequences among anammox bacteria. *Ca.* Loosdrechtia alkalitolerans is highlighted in red.

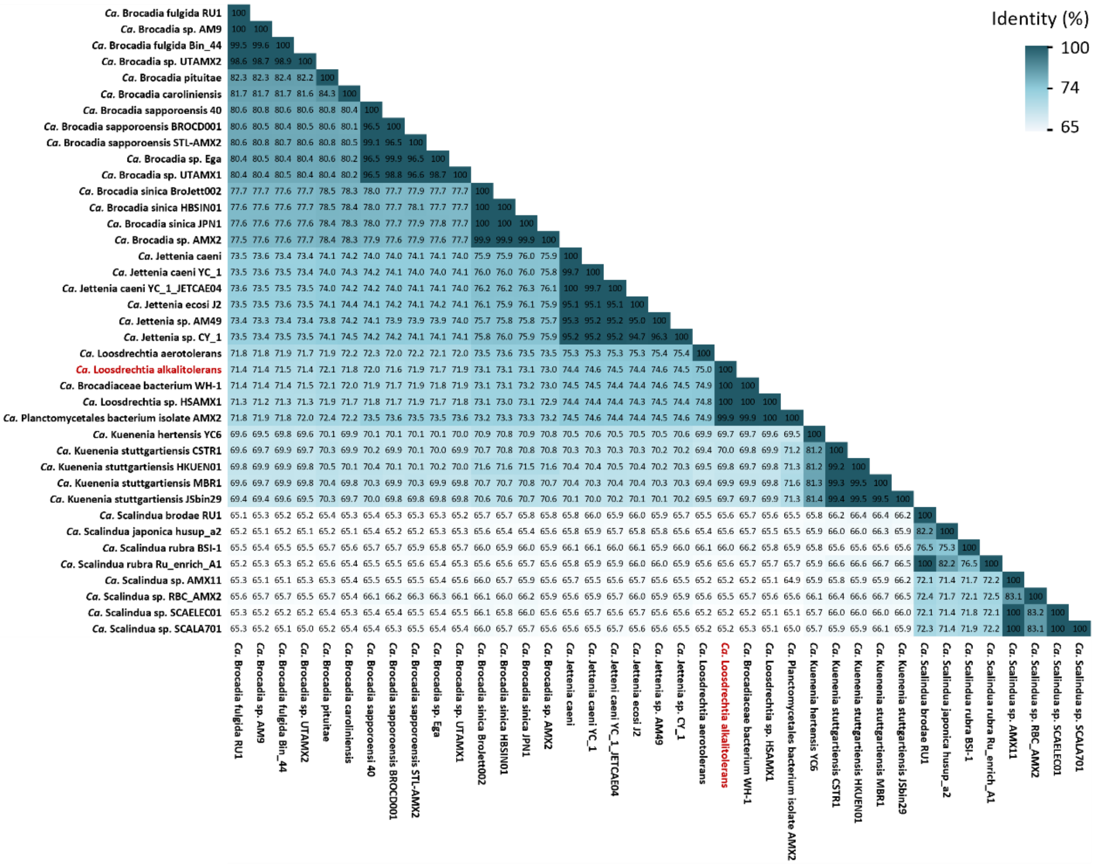

Figure S7. Pairwise orthologous average nucleotide identity (OrthoANI) matrix among anammox MAGs. *Ca.* Loosdrechtia alkalitolerans is highlighted in red.

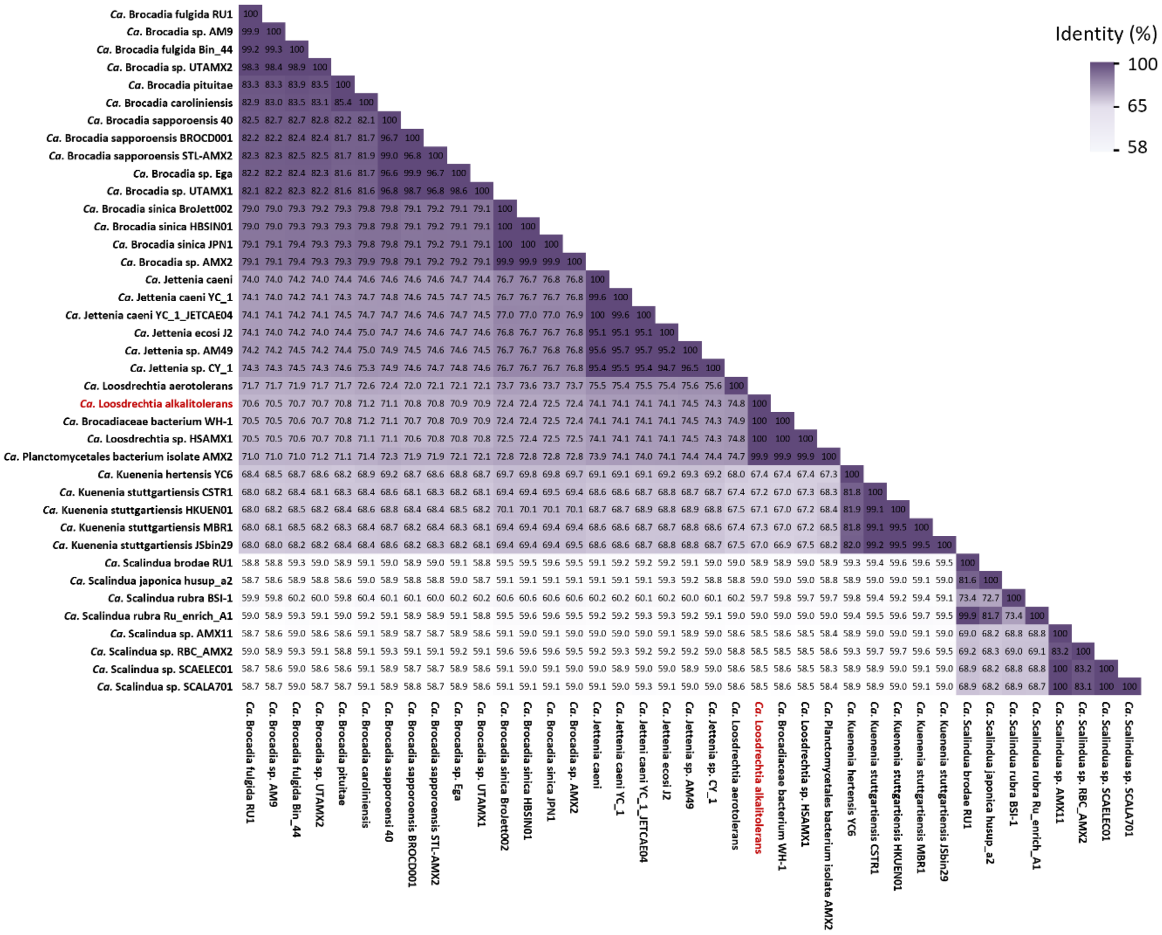

Figure S8. Pairwise average amino acid identity (AAI) matrix among anammox MAGs. *Ca.* Loosdrechtia alkalitolerans is highlighted in red.

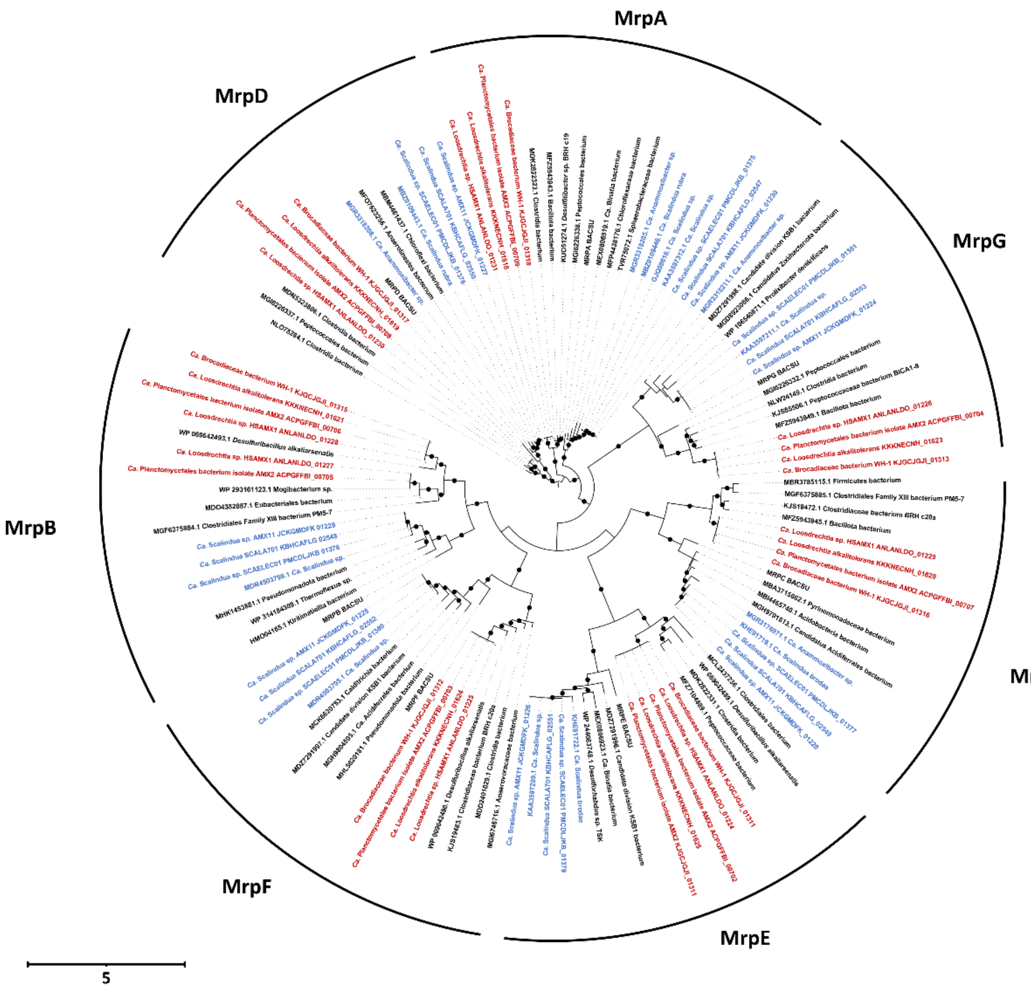

Figure S9. Phylogenetic analysis of Mrp complex subunits in *Ca*. Loosdrechtia alkalitolerans and marine anammox genera. Reference sequences were retrieved from the NCBI ClusteredNR database by blastp searches using Mrp sequences from *Ca.* Loosdrechtia alkalitolerans and *Ca.* Scalindua spp. as seed queries. All Mrp homologs were aligned with MAFFT using the L-INS-i option, and trimmed with trimAl using the -automated1 option. A maximum-likelihood tree was inferred with IQ-TREE3 under the best-fit model LG+F+G4 selected by ModelFinder, with 1,000 ultrafast bootstrap replicates; bootstrap values >70 are shown in soild circle. The scale bar shows the estimated number of substitutions per site. Sequences closely related to *Ca.* Loosdrechtia alkalitolerans and marine anammox genera (*Ca*. Scalindua and *Ca*. Anammoxibacter) are highlighted in red and blue, respectively.

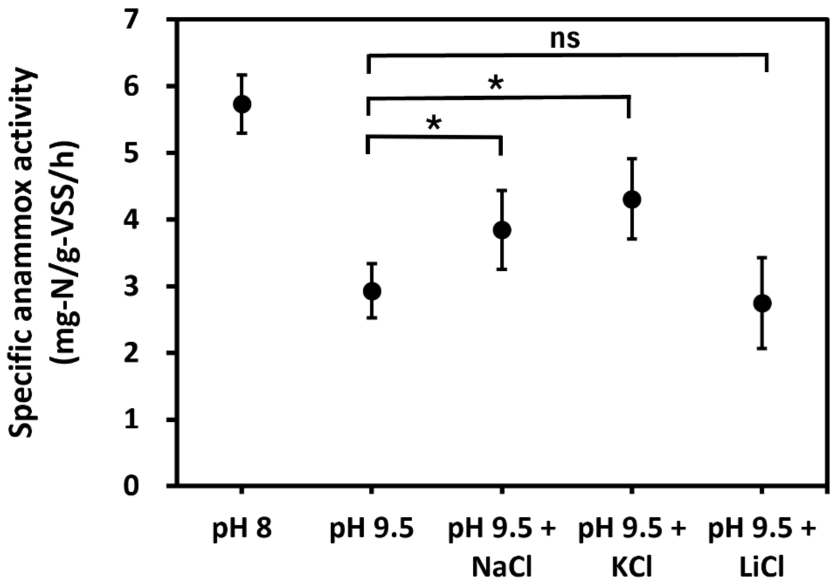

Figure S10. Cation-specific recovery of specific anammox activity (SAA) under alkaline stress. Biomass harvested from the KJ reactor on day 982 was used in this experiment. Because the reactor was operated at a higher pH during this period (8.92 ± 0.11) compared to the biomass used for the batch assays presented in the main text (8.52 ± 0.14), the sludge may have adapted to more alkaline conditions. Therefore, pH 9.5, which caused ~50% inhibition of anammox activity, was used as the alkaline control. Na^+, K^+, and Li^+ were added at a final concentration of 92.7 mM, comparable to the condition used in Figure 6. SAA was calculated from the linear regression of the total ammonia nitrogen (NH_3+NH_4^+) depletion profile ($R^2 > 0.913$) and normalized to VSS. Statistical significance was determined using Student's *t* test (*$P < 0.05$). "ns" indicates not significant. Error bars represent the standard deviation from three biological replicates.

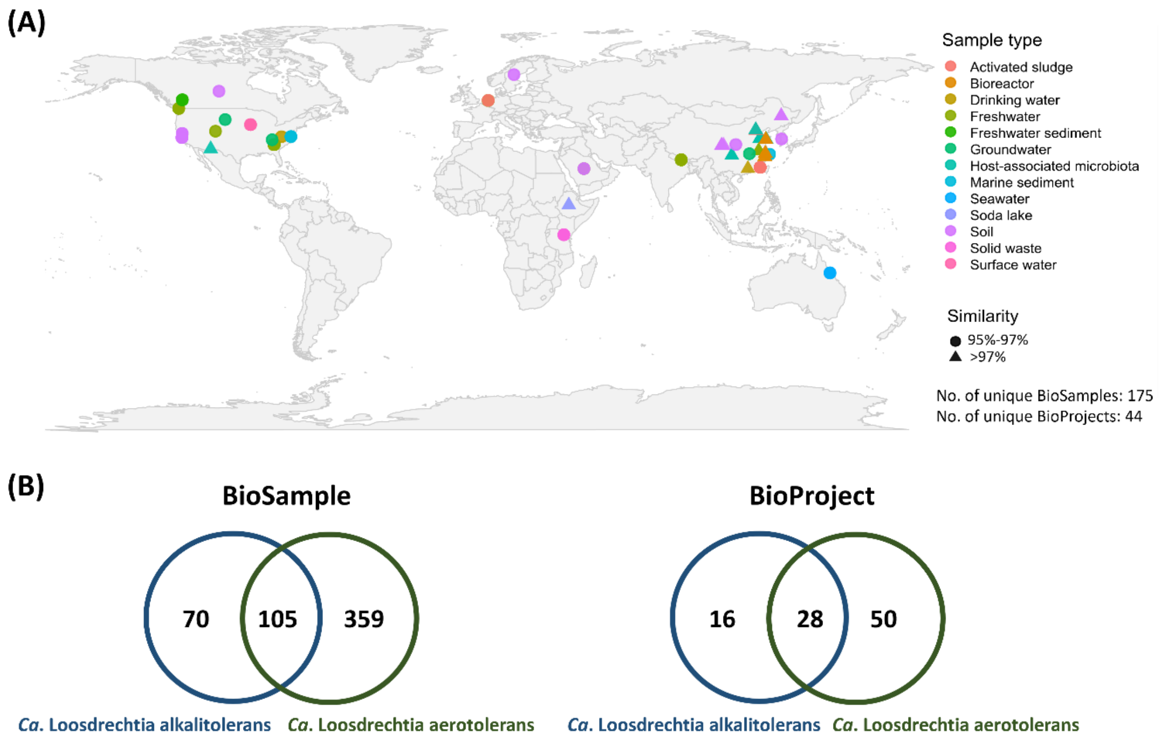

Figure S11. Global occurrence and environmental overlap of *Ca.* Loosdrechtia species. (A) Global occurrence of *Ca.* Loosdrechtia alkalitolerans-affiliated sequences in public IMNGS datasets. **(B)** Venn diagrams illustrating the overlap between IMNGS hits retrieved using the full-length 16S rRNA genes of *Ca*. Loosdrechtia alkalitolerans and *Ca*. Loosdrechtia aerotolerans as queries. The overlap is summarized at the BioSample level (left) and BioProject level (right). Numbers indicate the counts of shared and unique BioSamples and BioProjects.

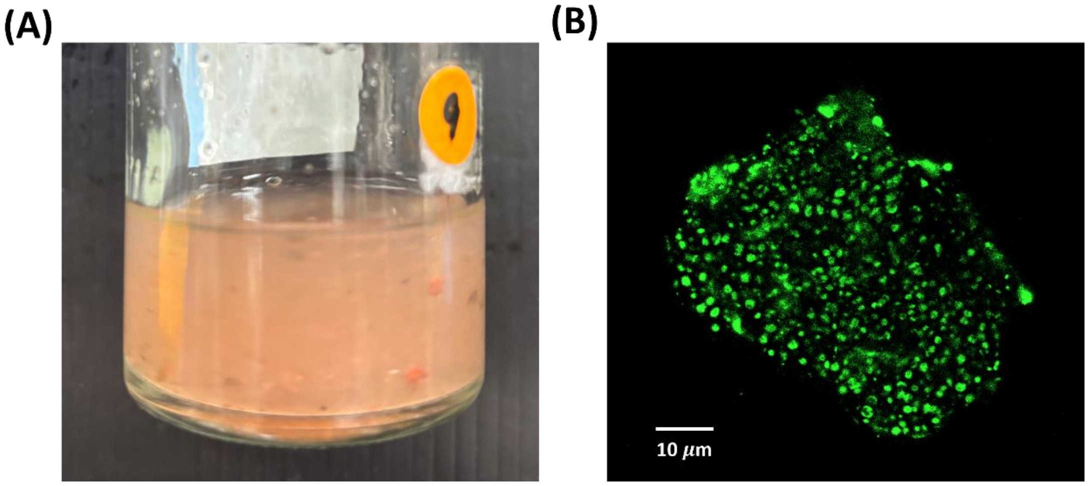

Figure S12. ***Ca.*** **Loosdrechtia alkalitolerans-dominant biomass enriched in this study. (A)** Appearance of the harvested biomass. **(B)** Confocal laser scanning microscopy image of the harvested biomass. Cells were hybridized using the 6-FAM-labeled Amx368 probe (targeting all anammox bacteria) in a hybridization buffer containing 15% formamide.

References

1. Rice EW, Bridgewater L, Association APH. *Standard methods for the examination of water and wastewater*, 22nd edn. Washington, DC: American Public Health Association, 2012.
2. Ye J, Coulouris G, Zaretskaya I *et al.* Primer-BLAST: a tool to design target-specific primers for polymerase chain reaction. *BMC Bioinformatics*. 2012;**13**:1-11 https://doi.org/10.1186/1471-2105-13-134
3. Chuang H-Y, Chen W-Y, Chen S-H *et al.* Metagenome-assembled genome sequence of *Candidatus* Loosdrechtia sp. KJ reconstructed from an alkaline anammox reactor. *Microbiol Resour Announc*. 2025;**14**:e01024-25 https://doi.org/10.1128/mra.01024-25
4. Bolyen E, Rideout JR, Dillon MR *et al.* Reproducible, interactive, scalable and extensible microbiome data science using QIIME 2. *Nat Biotechnol*. 2019;**37**:852-57 https://doi.org/10.1038/s41587-019-0209-9
5. Callahan BJ, McMurdie PJ, Rosen MJ *et al.* DADA2: High-resolution sample inference from Illumina amplicon data. *Nat Methods*. 2016;**13**:581-83 https://doi.org/10.1038/nmeth.3869
6. Bokulich NA, Kaehler BD, Rideout JR *et al.* Optimizing taxonomic classification of marker-gene amplicon sequences with QIIME 2's q2-feature-classifier plugin. *Microbiome*. 2018;**6**:90 https://doi.org/10.1186/s40168-018-0470-z
7. Lagkouvardos I, Joseph D, Kapfhammer M *et al.* IMNGS: a comprehensive open resource of processed 16S rRNA microbial profiles for ecology and diversity studies. *Sci Rep*. 2016;**6**:33721 https://doi.org/10.1038/srep33721
8. Yarza P, Yilmaz P, Pruesse E *et al.* Uniting the classification of cultured and uncultured bacteria and archaea using 16S rRNA gene sequences. *Nat Rev Microbiol*. 2014;**12**:635-45 https://doi.org/10.1038/nrmicro3330
9. Egli K, Fanger U, Alvarez PJ *et al.* Enrichment and characterization of an anammox bacterium from a rotating biological contactor treating ammonium-rich leachate. *Arch Microbiol*. 2001;**175**:198-207 https://doi.org/10.1007/s002030100255
10. Okabe S, Kamizono A, Zhang L *et al.* Salinity tolerance and osmoadaptation strategies in four genera of anammox bacteria: *Brocadia*, *Jettenia*, *Kuenenia*, and *Scalindua*. *Environ Sci Technol*. 2024;**58**:5357-71 https://doi.org/10.1021/acs.est.3c07324
11. Oshiki M, Shimokawa M, Fujii N *et al.* Physiological characteristics of the anaerobic ammonium-oxidizing bacterium '*Candidatus* Brocadia sinica'.

Microbiology. 2011;**157**:1706-13 https://doi.org/10.1099/mic.0.048595-0

12. Ali M, Oshiki M, Awata T *et al.* Physiological characterization of anaerobic ammonium oxidizing bacterium '*Candidatus* Jettenia caeni'. *Environ Microbiol*. 2015;**17**:2172-89 https://doi.org/10.1111/1462-2920.12674
13. Awata T, Oshiki M, Kindaichi T *et al.* Physiological characterization of an anaerobic ammonium-oxidizing bacterium belonging to the "*Candidatus* Scalindua" group. *Appl Environ Microbiol*. 2013;**79**:4145-48 https://doi.org/10.1128/AEM.00056-13
14. Anthonisen AC, Loehr RC, Prakasam T *et al.* Inhibition of nitrification by ammonia and nitrous acid. *J Water Pollut Control Fed*. 1976;**48**:835-52
